# Dynamic Behavior of Magnetically Affected Rod-Like Nanostructures with Multiple Defects via Nonlocal-Integral/Differential-Based Models

**DOI:** 10.3390/nano10112306

**Published:** 2020-11-21

**Authors:** Keivan Kiani, Krzysztof Kamil Żur

**Affiliations:** 1 Department of Civil Engineering, K.N. Toosi University of Technology, Valiasr Ave., P.O. Box 15875-4416, Tehran, Iran; k_kiani@kntu.ac.ir; 2 Faculty of Mechanical Engineering, Bialystok University of Technology, Wiejska 45C Street, 15-351 Bialystok, Poland

**Keywords:** defected nanorods, longitudinal free vibration, bilaterally applied magnetic field, nonlocal-integro-based model, surface energy effect, finite-element method (FEM)

## Abstract

Through considering both nonlocality and surface energy effects, this paper suggests suitable mathematical-continuum-based models for free vibration of nanorods with multiple defects acted upon by a bidirectional-transverse magnetic field. By employing both theories of elasticity of Eringen and Gurtin–Murdoch, the equations of motion for the magnetically affected-damaged rod-like nanostructures are derived using the nonlocal-differential-based and the nonlocal-integral-based models. The local defects are modeled by a set of linearly appropriate axial springs at the interface of appropriately divided nanorods. Through constructing the nonlocal-differential equations of motion for sub-divided portions and by imposing the appropriate interface conditions, the natural frequencies as well as the vibrational modes are explicitly obtained for fixed–free and fixed–fixed nanorods with low numbers of defects. The extracted nonlocal-integral governing equations are also solved for natural frequencies using the finite-element technique. For a particular situation, the model’s results are successfully verified with those of another work. Subsequently, the effects of nonlocality, surface energy, defect’s location, nanorod’s diameter, magnetic field strength, and number of defects on the dominant free vibration response of the magnetically defected nanorods with various end conditions are displayed and discussed.

## 1. Introduction

Nanorods represent a special morphology of nanoscale objects whose sizes range from 1 to 100 nm and their lateral dimensions are fairly negligible in comparison to their lengths. They are commonly created by chemical synthesis, including cation exchange [1,2,3] and hydrothermal method [4,5]. Nanorods would have great applications in display technologies [6,7], micro-electro-mechanical systems [8,9,10,11], cancer therapy [12,13], energy harvesting [14,15], light-emitting devices [16,17], and solar cells [18,19,20]. Any defect in nanorods during their synthesis or after that may limit their applications. As a result, recognizing the mechanical behavior of defected nanorods would greatly expand our basic understanding about their mechanical limit and optimal design for the considered duty.

From applied mechanics point of view, the main structural mechanic characteristic of nanorods is the axial rigidity. At the nanoscale, nonlocality and the potential surface energy are among the crucial factors that discriminate the nature of the axial rigidity from that at the macroscale. If a nanorod is defected by an external agent in one limited zone or several distinct points, one confronts to a locally defected nanorod whose axial rigidity as well as axial stiffness at the defected points or zones would be lower than those of the intact zones and depends on the damage level. As a result, the assessment of the mechanical behavior of defected nanorods is of great importance. This study concerns with the role of local defects on the axial vibration of nanorods by considering the nonlocality as well as the surface influence.

In nanorod structures, each atom’s vibration could influence its adjacent atoms’ vibrations due to the existence of the inter-atomic bonds. In other words, the stress status at a point depends on the statuses of stress at the contiguous points. This important vision of the stress field cannot be captured and displayed by the traditional elasticity theory (TET). To vanquish this shortcoming, nonlocal elasticity theory (NET) was developed by Eringen [21,22]. To date, the NET of Eringen has been vastly utilized for mechanical analysis of rod-like [23,24,25,26,27,28], beam-like [29,30,31,32,33,34,35,36,37,38], and plate-like [39,40,41,42,43] nanostructures. Recently, the nonlocal stress-driven formulations have been established for thermoelastic analysis of straight nanobeams as well as elastostatics of curved ones [44,45] to overcome the ill-posed nonlocal-differential-based models under certain situations. In another perspective, configuration of electron gases in core-shell-based nanowires was examined using a nonlocal pseudospectral approach [46]. Actually, this research work recasts a nonlocal term into a separate differential equation that must be solved simultaneously as a system of partial differential equations. On the other hand, at the nanoscale, the strain energy of the surface layer could not be neglected due to the fairly high ratio of the surface area to the bulk volume. Gurtin and Murdoch [47,48,49,50] suggested a surface continuum-based theory to explain the influence of the surface energy on the overall mechanical response of structures at the nanoscale. Based on their suggested model, at the low-dimensional scales, the whole body of the structure could be imagined consisting of two crucial parts: surface layer (SL) and the bulk zone (BZ). The constitution relations of the BZ are the same as those of macroscale structures in the absence of the nonlocality. Further, the surface elasticity theory (SET) displays that the strain–stress relations of the SL are dissimilar to those of the BZ [51]. The constants of these relations (i.e., Lame’s constants of the SL plus to the initial stress at unstrained conditions) could be evaluated by comparing the predicted SET-based results and those of a suitable atomic model. Until now, the SET has been adopted to many problems associated with the mechanics of nanostructures [52,53,54,55,56,57,58,59,60]. For more rational modeling of nanostructures, mixed-formulations of the NET by Eringen and the SET by Gurtin and Murdoch, the so-called nonlocal surface elasticity theory (NSET), should be employed. There exist several works on the application of the NSET to mechanical problems of nanostructures such as nanorods [61,62], nanobeams [63,64,65,66,67], and nanoplates [68,69,70,71]. Further, modeling of rod-like and beam-like nanostructures based on the nonlocal strain elasticity theory has been the subject of various research works in recent years [72,73,74]; however, vibrations of nanorods with multiple defects in the presence of both lateral and transverse magnetic fields have not been addressed in the context of the NSET.

In highly conducting materials, the exertion of an appropriately oriented magnetic field would generally change the whole stiffness (i.e., the sum of the internal, external, and geometrical stiffness values) of the nanostructure in desired directions. Thereby, the application of a magnetic field would effectively influence on the characteristics of sound waves propagate within them as well as their vibrations in particular directions. So far, vibrations of nonlocal nanobeams [75,76,77], nanoplates [78,79], and nanorods [80,81] under various actions of magnetic fields have been scrutinized in some details. All of these magnetically affected nanostructures were defect-free. This brief literature survey shows that the influence of the magnetic field on defected nanostructures has not been methodically explained.

The present paper has been organized as follows: in Section 2, the nonlocal-differential-based relations of the longitudinal motion for locally damaged nanorods, acted upon by an arbitrarily transverse magnetic field as well as the governing interfacial conditions, are displayed—the so-called nonlocal-differential-surface energy-based model (NDSM). For the magnetically affected nanostructure with fixed–free and fully fixed ends, the dispersion relation associated with longitudinal vibrations are explicitly derived, and the vibration modes pertinent to each boundary condition are computed for the case of a single defect. In Section 3, the nonlocal-integral governing equations of the magnetically affected nanorod with multiple defects are explained; the so-called nonlocal-integral-surface energy-based model (NISM). Then, a finite-element method (FEM) is suggested for its frequency analysis. In Section 4, a comparison study as well as a fairly inclusive parametric study is given, and the roles of crucial factors on the axial vibration of magnetically affected rod-like nanostructures with multiple defects and different end conditions are discussed in some details.

## 2. Establishing an NDSM for the Magnetically Affected Defected Nanorods

### 2.1. Problem Delineation

**Figure 1:**
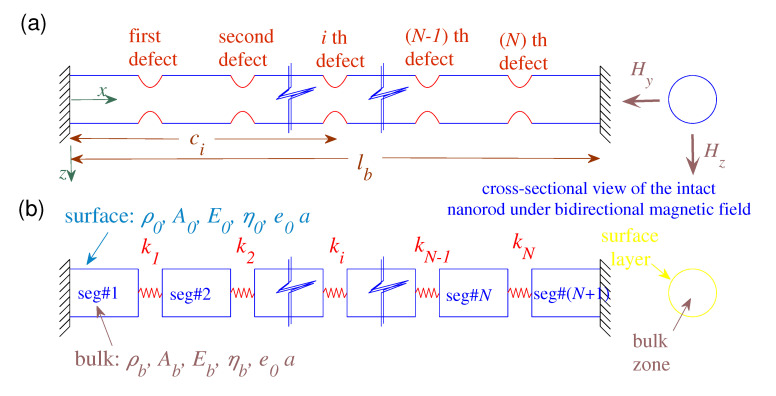
(**a**) A nanorod with multiple defects acted upon by bilateral magnetic fields with fixed–fixed ends. (**b**) Schematically represented continuum-based defected nanorod.

Consider a magnetically influenced nanorod with multiple defects as depicted schematically in Figure 1a. The length and the diameter of the nanorod are denoted by $l_{b}$ and $D_{0}$, respectively, and the locations of the localized defects from the left end are symbolized by $c_{i}$ where i=1,2,…,N. The defects are configured in an axisymmetric manner and the their lengths would be almost slight in comparison with the length of the nanostructure. The nanorod is assumed to be uniform (except at the defected zones), homogeneous, isotropic, and highly conductive. The defected nanorod is under action of a transversely oblique magnetic field whose strength vector is given by: $H=H_{y}\;e_{y}+H_{z}\;e_{z}$ where $e_{y}$ and $e_{z}$ represent the unit base vectors along the *y*-axis and *z*-axis, respectively, and $H_{y}$ and $H_{z}$ in order are the components of the magnetic field strength along the *y* and *z* axes (see Figure 1a). Since the defects are axisymmetric and locally placed along the length of the nanorod, they could be appropriately modeled by axial springs of constant $k_{i}$, as shown in Figure 1b. The magnitude of $k_{i}$ for the *i*th defect mainly depends on the geometry of the defected zone, the mechanical properties of the BZ and the SL of the defect zone, and the transverse strengths of the magnetic field.

### 2.2. Construction of Nonlocal-Differential-Surface-Based Governing Equations

#### 2.2.1. Preliminaries

On the basis of the theory of elastic bodies of Gurtin and Murdoch [47,48,49], the SL is a skinny layer attached strongly to its underlying BZ. Herein, the longitudinal displacement and the corresponding strain of both SL and BZ in order are denoted by u=u(x,t) and $\varepsilon =\frac{\partial u}{\partial x}$. The physical and mechanical properties of the SL are basically unlike those of the BZ. These properties are typically assessed by comparing the surface-energetic mechanical behavior of fairly long nanorods and that predicted by an appropriate atomic model. The Young’s modulus, density, and the cross-section area of the BZ are $E_{b}$, $\rho_{b}$, and $A_{b}$ while the elastic modulus, density, and cross-sectional pyramid of the SL are represented by $E_{0}$, $\rho_{0}$, and $A_{0}$, respectively. The only nonzero local stress within the BZ and the SL in order are given by: $\sigma_{bx}^{l}=E_{b}\varepsilon_{x}$ and $\sigma_{sx}^{l}=E_{0}\varepsilon_{x}+\tau_{0}$, where $\tau_{0}$ is the residual stress within the SL. On the other hand, these are local stresses which would be valid for nanorods acted upon by longitudinal waves with fairly long wavelengths. If the longitudinal vibration occurs due to the waves of wavelengths comparable with the inter-atom bonds, the nonlocality becomes important. According to the nonlocal continuum theory of Eringen [21,22], the nonlocal elastic stresses at a point of the SL or the BZ could be related to the local ones by the following constitutive relation: $\sigma_{\alpha x}^{nl}-{\left(e_{0}a\right)}^{2}\frac{\partial^{2}}{\partial x^{2}}\sigma_{\alpha x}^{nl}=\sigma_{\alpha x}^{l}$, where α=bors, and $e_{0}a$ denotes the small-scale parameter. Through integrating of both sides of the recent relation over the cross-sectional domain of the nanorod and then adding the resulted expressions, it is obtainable:

(1) $$\begin{array}{c} N^{nl}-{\left(e_{0}a\right)}^{2}\frac{\partial^{2}N^{nl}}{\partial x^{2}}=N^{cl}, \end{array}$$

where $N^{cl}$ and $N^{nl}$ are the local and nonlocal axial forces. In the following part, we proceed in evaluating the local axial force within the magnetically affected nanorods with the surface effect.

#### 2.2.2. Classical Axial Force within a Magnetically Influenced Nanorod

Since the evaluation of only the local axial force of the magnetically affected nanorod is of special concern in this part, the small-scale parameter is set equal to zero. Hence, the requirement of dynamic equilibrium for an infinitesimal element of the magnetically affected nanorod of length *dx* by considering the inertia of the SL leads to:

(2) $$\begin{array}{c} \frac{\partial N}{\partial x}+f_{mx}=\left(\rho_{b}A_{b}+\rho_{0}A_{0}\right)\frac{\partial^{2}u}{\partial t^{2}}, \end{array}$$

where N=N(x,t) denotes the dynamical axial force field within the nanorod in the absence of the magnetic field, and $f_{mx}$ represents the magnetic force per unit length of the nanorod. In the framework of linear surface elasticity theory of Gurtin and Murdoch [47,48], the aforementioned axial force is stated by:

(3) $$\begin{array}{c} N=\left(E_{b}A_{b}+E_{0}A_{0}\right)\frac{\partial u}{\partial x}+\tau_{0}S_{0}, \end{array}$$

in which $S_{0}$ is the length of the cross-section of the SL. From the structural engineering standpoint, $E_{b}A_{b}$ and $E_{0}A_{0}$ represent the axial rigidity of the BZ and the SL, respectively, and $\tau_{0}S_{0}$ is the resulted axial force within the nanorod (compressive or tensile force depend on the sign of $\tau_{0}$).

By using Ref. [76], the magnetic field force applied on both SL and BZ of the highly conducting nanorod is evaluated by:

(4a) $$\begin{array}{c} \begin{array}{c} f_{ms}=\eta_{0}\;\left(\nabla \times \left(\nabla \times \left(u\times H\right)\right)\right)\times H, \end{array} \end{array}$$

(4b) $$\begin{array}{c} \begin{array}{c} f_{mb}=\eta_{b}\;\left(\nabla \times \left(\nabla \times \left(u\times H\right)\right)\right)\times H, \end{array} \end{array}$$

where ∇ is the gradient symbol, H denotes the strength of the magnetic field vector, $\eta_{0}$ and $\eta_{b}$ represent the magnetic permeability of the SL and the BZ, respectively, and u denotes the vector of displacement field. By introducing $u=u\left(x,t\right)e_{x}$ and $H=H_{x}\;e_{x}+H_{y}\;e_{y}+H_{z}\;e_{z}$ to Equation (4), the only non-vanishing component of the exerted magnetic field on the nanorod is derived as:

(5) $$\begin{array}{c} f_{mx}=\left(\eta_{0}A_{0}+\eta_{b}A_{b}\right)\;\left(H_{y}^{2}+H_{z}^{2}\right)\;\frac{\partial^{2}u}{\partial x^{2}}. \end{array}$$

Now, Equations (3) and (5) are substituted into Equation (2) to result in the local equation of motion accounting for the surface energy effect:

(6) $$\begin{array}{c} \frac{\partial N^{cl}}{\partial x}=\left(\rho_{b}A_{b}+\rho_{0}A_{0}\right)\frac{\partial^{2}u}{\partial t^{2}}, \end{array}$$

where

(7) $$\begin{array}{c} N^{cl}=\left[\left(E_{b}A_{b}+E_{0}A_{0}\right)+\left(\eta_{0}A_{0}+\eta_{b}A_{b}\right)\;\left(H_{y}^{2}+H_{z}^{2}\right)\right]\;\frac{\partial u}{\partial x}+\tau_{0}S_{0}. \end{array}$$

Equation (7) furnishes us with the classical axial force within a magnetically affected nanorod. In the case of $E_{0}>0$, both SL and applied transverse magnetic field tend to increase the nanorod’s axial rigidity. It is noticed that the residual surface axial force does not incorporate to the axial rigidity of the nanostructure; however, it results in initial static deformation within the defected nanorod. Such an axial internal load could have influence the nanostructure’s transverse vibration, which is not of concern of authors in the present work.

### 2.3. Equations of Motion Associated with the NDSM

By virtue of Equation (2), the equation of motion as a function of the nonlocal axial force in the absence of body force takes the following form:

(8) $$\begin{array}{c} \frac{\partial N^{nl}}{\partial x}=\left(\rho_{b}A_{b}+\rho_{0}A_{0}\right)\frac{\partial^{2}u}{\partial t^{2}}, \end{array}$$

where the nonlocal axial force is governed by the following equation through introducing Equation (7) to Equation (1),

(9) $$\begin{array}{c} N^{nl}-{\left(e_{0}a\right)}^{2}\frac{\partial^{2}N^{nl}}{\partial x^{2}}=\left[\left(E_{b}A_{b}+E_{0}A_{0}\right)+\left(\eta_{0}A_{0}+\eta_{b}A_{b}\right)\;\left(H_{y}^{2}+H_{z}^{2}\right)\right]\;\frac{\partial u}{\partial x}+\tau_{0}S_{0}. \end{array}$$

By mixing Equation (8) and Equation (9), the nonlocal axial force within the defect-free nanorod under a transverse magnetic field is written by:

(10) $$\begin{array}{cc} N^{nl}= & \left[\left(E_{b}A_{b}+E_{0}A_{0}\right)+\left(\eta_{0}A_{0}+\eta_{b}A_{b}\right)\;\left(H_{y}^{2}+H_{z}^{2}\right)\right]\;\frac{\partial u}{\partial x}\;\\ & +{\left(e_{0}a\right)}^{2}\left(\rho_{b}A_{b}+\rho_{0}A_{0}\right)\frac{\partial^{3}u}{\partial t^{2}\partial x}+\tau_{0}S_{0}, \end{array}$$

and the nonlocal-surface energy-based governing equation is derived as follows:

(11) $$\begin{array}{c} \left[\left(E_{b}A_{b}+E_{0}A_{0}\right)+\left(\eta_{0}A_{0}+\eta_{b}A_{b}\right)\;\left(H_{y}^{2}+H_{z}^{2}\right)\right]\;\frac{\partial^{2}u}{\partial x^{2}}=\left(\rho_{b}A_{b}+\rho_{0}A_{0}\right)\left[1-{\left(e_{0}a\right)}^{2}\frac{\partial^{2}}{\partial x^{2}}\right]\frac{\partial^{2}u}{\partial t^{2}}. \end{array}$$

For a nanorod with *N* locally defected zones (see Figure 1b), the magnetically affected nanostructure is divided to N+1 distinct parts (i.e., segments) such that the longitudinal dynamic displacement of the *i*th segment are represented by $u_{i}=u_{i}\left(x,t\right)$. Using Equation (11), the governing equations of the first, intermediate, and last segments would be:

(12a) $$\begin{array}{c} \begin{array}{c} \left[\left(E_{b}A_{b}+E_{0}A_{0}\right)+\left(\eta_{0}A_{0}+\eta_{b}A_{b}\right)\;\left(H_{y}^{2}+H_{z}^{2}\right)\right]\frac{\partial^{2}u_{1}}{\partial x^{2}}=\;\\ \left(\rho_{b}A_{b}+\rho_{0}A_{0}\right)\left[1-{\left(e_{0}a\right)}^{2}\frac{\partial^{2}}{\partial x^{2}}\right]\frac{\partial^{2}u_{1}}{\partial t^{2}};\;\;0<x<c_{1}, \end{array} \end{array}$$

(12b) $$\begin{array}{c} \begin{array}{c} \left[\left(E_{b}A_{b}+E_{0}A_{0}\right)+\left(\eta_{0}A_{0}+\eta_{b}A_{b}\right)\;\left(H_{y}^{2}+H_{z}^{2}\right)\right]\frac{\partial^{2}u_{i}}{\partial x^{2}}=\;\\ \left(\rho_{b}A_{b}+\rho_{0}A_{0}\right)\left[1-{\left(e_{0}a\right)}^{2}\frac{\partial^{2}}{\partial x^{2}}\right]\frac{\partial^{2}u_{i}}{\partial t^{2}};\;\;c_{i-1}<x<c_{i}, \end{array} \end{array}$$

(12c) $$\begin{array}{c} \begin{array}{c} \left[\left(E_{b}A_{b}+E_{0}A_{0}\right)+\left(\eta_{0}A_{0}+\eta_{b}A_{b}\right)\;\left(H_{y}^{2}+H_{z}^{2}\right)\right]\frac{\partial^{2}u_{N+1}}{\partial x^{2}}=\;\\ \left(\rho_{b}A_{b}+\rho_{0}A_{0}\right)\left[1-{\left(e_{0}a\right)}^{2}\frac{\partial^{2}}{\partial x^{2}}\right]\frac{\partial^{2}u_{N+1}}{\partial t^{2}};\;\;c_{N}<x<1, \end{array} \end{array}$$

where i=2,3,…,N.

By modeling of the *i*th locally defect zone by an axial spring of constant $k_{i}$, the continuity of the nonlocal axial force at the interface yields the following interface conditions:

(13a) $$\begin{array}{c} \begin{array}{cc} k_{i}\left[u_{i+1}\left(c_{i},t\right)-u_{i}\left(c_{i},t\right)\right]= & \left[\left(E_{b}A_{b}+E_{0}A_{0}\right)+\left(\eta_{0}A_{0}+\eta_{b}A_{b}\right)\;\left(H_{y}^{2}+H_{z}^{2}\right)+\right.\;\\ & \left.{\left(e_{0}a\right)}^{2}\left(\rho_{b}A_{b}+\rho_{0}A_{0}\right)\frac{\partial^{2}}{\partial t^{2}}\right]\frac{\partial u_{i}}{\partial x}\left(c_{i},t\right);\;i=1,2,\ldots ,N, \end{array} \end{array}$$

(13b) $$\begin{array}{c} \begin{array}{c} \left[\left(E_{b}A_{b}+E_{0}A_{0}\right)+\left(\eta_{0}A_{0}+\eta_{b}A_{b}\right)\;\left(H_{y}^{2}+H_{z}^{2}\right)+{\left(e_{0}a\right)}^{2}\left(\rho_{b}A_{b}+\rho_{0}A_{0}\right)\frac{\partial^{2}}{\partial t^{2}}\right]\frac{\partial u_{i}}{\partial x}\left(c_{i},t\right)=\;\\ \left[\left(E_{b}A_{b}+E_{0}A_{0}\right)+\left(\eta_{0}A_{0}+\eta_{b}A_{b}\right)\;\left(H_{y}^{2}+H_{z}^{2}\right)+{\left(e_{0}a\right)}^{2}\left(\rho_{b}A_{b}+\rho_{0}A_{0}\right)\frac{\partial^{2}}{\partial t^{2}}\right]\frac{\partial u_{i+1}}{\partial x}\left(c_{i},t\right). \end{array} \end{array}$$

The boundary conditions of the fixed–fixed (FIFI) and the fixed–free (FIFR) damaged nanorods are expressed by:

(14a) $$\begin{array}{c} \begin{array}{c} \mathrm{fixed}-\mathrm{fixed}\;(\mathrm{FIFI}):\;\;u_{1}\left(0,t\right)=0,\;\;u_{N+1}\left(l_{b},t\right)=0, \end{array} \end{array}$$

(14b) $$\begin{array}{c} \begin{array}{c} \mathrm{fixed}-\mathrm{free}\;(\mathrm{FIFR}):\left\{\begin{array}{c} u_{1}\left(0,t\right)=0,\;\\ \left[\begin{array}{c} \left(E_{b}A_{b}+E_{0}A_{0}\right)+{\left(e_{0}a\right)}^{2}\left(\rho_{b}A_{b}+\rho_{0}A_{0}\right)\frac{\partial^{2}}{\partial t^{2}}\\ +\left(\eta_{0}A_{0}+\eta_{b}A_{b}\right)\;\left(H_{y}^{2}+H_{z}^{2}\right) \end{array}\right]\;\frac{\partial u_{N+1}}{\partial x}\left(l_{b},t\right)=0 \end{array}.\right. \end{array} \end{array}$$

For the sake of convenience in the dynamical analysis of the nanomechanical problem, the following dimensionless factors are introduced:

(15) $$\begin{array}{c} \xi =\frac{x}{l_{b}},\;\;{\bar{u}}_{1}=\frac{u_{1}}{l_{b}},\;\;{\bar{u}}_{2}=\frac{u_{2}}{l_{b}},\;\;{\bar{c}}_{i}=\frac{c_{i}}{l_{b}},\;\;\mu =\frac{e_{0}a}{l_{b}},\;\;\tau =\frac{t}{l_{b}}\sqrt{\frac{E_{b}}{\rho_{b}}},\;{\bar{k}}_{i}=\frac{k_{i}l_{b}}{E_{b}A_{b}},\;\\ \chi_{1}^{2}=\frac{\rho_{0}A_{0}}{\rho_{b}A_{b}},\;\chi_{2}^{2}=\frac{E_{0}A_{0}}{E_{b}A_{b}},\;\chi_{3}^{2}=\frac{\eta_{0}A_{0}}{\eta_{b}A_{b}},\;{\bar{H}}_{y}=H_{y}\;\sqrt{\frac{\eta_{b}}{E_{b}}},\;{\bar{H}}_{z}=H_{z}\;\sqrt{\frac{\eta_{b}}{E_{b}}}. \end{array}$$

where ${\bar{k}}_{i}$ is the dimensionless damage factor of the *i*th defect. By introducing Equation (15) to Equations (12a)–(12c), the dimensionless equations of motion of various portions of the damaged nanorod take the following form:

(16a) $$\begin{array}{c} \begin{array}{c} \left(1+\chi_{1}^{2}\right)\left(\frac{\partial^{2}{\bar{u}}_{1}}{\partial \tau^{2}}-\mu^{2}\frac{\partial^{4}{\bar{u}}_{i}}{\partial \tau^{2}\partial \xi^{2}}\right)\;\\ -\left[\left(1+\chi_{2}^{2}\right)+\left(1+\chi_{3}^{2}\right)\left({\bar{H}}_{y}^{2}+{\bar{H}}_{z}^{2}\right)\right]\;\frac{\partial^{2}{\bar{u}}_{1}}{\partial \xi^{2}}=0;\;\;0<\xi <{\bar{c}}_{1}, \end{array} \end{array}$$

(16b) $$\begin{array}{c} \begin{array}{c} \left(1+\chi_{1}^{2}\right)\left(\frac{\partial^{2}{\bar{u}}_{i}}{\partial \tau^{2}}-\mu^{2}\frac{\partial^{4}{\bar{u}}_{i}}{\partial \tau^{2}\partial \xi^{2}}\right)\;\\ -\left[\left(1+\chi_{2}^{2}\right)+\left(1+\chi_{3}^{2}\right)\left({\bar{H}}_{y}^{2}+{\bar{H}}_{z}^{2}\right)\right]\;\frac{\partial^{2}{\bar{u}}_{i}}{\partial \xi^{2}}=0;\;\;{\bar{c}}_{i-1}<\xi <{\bar{c}}_{i};\;i=2,3,\ldots ,N, \end{array} \end{array}$$

(16c) $$\begin{array}{c} \begin{array}{c} \left(1+\chi_{1}^{2}\right)\left(\frac{\partial^{2}{\bar{u}}_{N+1}}{\partial \tau^{2}}-\mu^{2}\frac{\partial^{4}{\bar{u}}_{i}}{\partial \tau^{2}\partial \xi^{2}}\right)\;\\ -\left[\left(1+\chi_{2}^{2}\right)+\left(1+\chi_{3}^{2}\right)\left({\bar{H}}_{y}^{2}+{\bar{H}}_{z}^{2}\right)\right]\;\frac{\partial^{2}{\bar{u}}_{N+1}}{\partial \xi^{2}}=0;\;\;{\bar{c}}_{N}<\xi <1. \end{array} \end{array}$$

Furthermore, by introducing Equation (15) to Equations (13a) and (13b), the dimensionless boundary conditions of segments at their connection points take the following form:

(17a) $$\begin{array}{c} \begin{array}{cc} {\bar{k}}_{i}\left[{\bar{u}}_{i+1}\left({\bar{c}}_{i},\tau \right)-{\bar{u}}_{i}\left({\bar{c}}_{i},\tau \right)\right]= & \left[\left(1+\chi_{2}^{2}\right)+\left(1+\chi_{3}^{2}\right)\;\left({\bar{H}}_{y}^{2}+{\bar{H}}_{z}^{2}\right)+\right.\;\\ & \left.\mu^{2}\left(1+\chi_{1}^{2}\right)\frac{\partial^{2}}{\partial \tau^{2}}\right]\frac{\partial {\bar{u}}_{i}}{\partial \xi }\left({\bar{c}}_{i},\tau \right);\;\; \end{array} \end{array}$$

(17b) $$\begin{array}{c} \begin{array}{c} \left[\left(1+\chi_{2}^{2}\right)+\left(1+\chi_{3}^{2}\right)\;\left({\bar{H}}_{y}^{2}+{\bar{H}}_{z}^{2}\right)+\mu^{2}\left(1+\chi_{1}^{2}\right)\frac{\partial^{2}}{\partial \tau^{2}}\right]\frac{\partial {\bar{u}}_{i}}{\partial \xi }\left({\bar{c}}_{i},\tau \right)=\;\\ \left[\left(1+\chi_{2}^{2}\right)+\left(1+\chi_{3}^{2}\right)\;\left({\bar{H}}_{y}^{2}+{\bar{H}}_{z}^{2}\right)+\mu^{2}\left(1+\chi_{1}^{2}\right)\frac{\partial^{2}}{\partial \tau^{2}}\right]\frac{\partial {\bar{u}}_{i+1}}{\partial \xi }\left({\bar{c}}_{i},\tau \right), \end{array} \end{array}$$

where i=1,2,…,N. The dimensionless conditions pertinent to the FIFI and FIFR ends could be written through introducing Equation (15) to Equations (14a) and (14b) as follows:

(18a) $$\begin{array}{c} \begin{array}{c} \mathrm{FIFI}:\;\;{\bar{u}}_{1}\left(0,\tau \right)=0,\;\;{\bar{u}}_{N+1}\left(1,\tau \right)=0, \end{array} \end{array}$$

(18b) $$\begin{array}{c} \begin{array}{c} \mathrm{FIFR}:\;\;{\bar{u}}_{1}\left(0,\tau \right)=0,\;\;\left[\begin{array}{c} \left(1+\chi_{2}^{2}\right)+\mu^{2}\left(1+\chi_{1}^{2}\right)\frac{\partial^{2}}{\partial \tau^{2}}\\ +\left(1+\chi_{3}^{2}\right)\;\left({\bar{H}}_{y}^{2}+{\bar{H}}_{z}^{2}\right) \end{array}\right]\;\frac{\partial {\bar{u}}_{N+1}}{\partial \xi }\left(1,\tau \right)=0. \end{array} \end{array}$$

### 2.4. Frequency Analysis via a Semi-Analytical Methodology

For free vibration analysis of the derived governing equations, we consider the following harmonic versions of dimensionless longitudinal displacements:

(19) $$\begin{array}{c} {\bar{u}}_{i}\left(\xi ,\tau \right)={\bar{U}}_{i}\left(\xi \right)e^{i\varpi \;\tau };\;\;i=1,2,\ldots ,N+1, \end{array}$$

in which i = $\sqrt{-1}$, ϖ is the dimensionless frequency ($\varpi =\omega \;l_{b}\;\sqrt{\frac{\rho_{b}}{E_{b}}}$), and τ is the dimensionless time parameter. By substituting Equation (19) into Equations (16a) and (16b), the following second-order ordinary differential equations are attained:

(20a) $$\begin{array}{c} \begin{array}{c} \frac{d^{2}{\bar{U}}_{1}}{d\xi^{2}}+\frac{\left(1+\chi_{1}^{2}\right)\varpi^{2}}{\left(1+\chi_{2}^{2}\right)+\left(1+\chi_{3}^{2}\right)\left({\bar{H}}_{y}^{2}+{\bar{H}}_{z}^{2}\right)-\mu^{2}\varpi^{2}\left(1+\chi_{1}^{2}\right)}{\bar{U}}_{1}=0;\;\;\;0<\xi <{\bar{c}}_{1}, \end{array} \end{array}$$

(20b) $$\begin{array}{c} \begin{array}{c} \frac{d^{2}{\bar{U}}_{i}}{d\xi^{2}}+\frac{\left(1+\chi_{1}^{2}\right)\varpi^{2}}{\left(1+\chi_{2}^{2}\right)+\left(1+\chi_{3}^{2}\right)\left({\bar{H}}_{y}^{2}+{\bar{H}}_{z}^{2}\right)-\mu^{2}\varpi^{2}\left(1+\chi_{1}^{2}\right)}{\bar{U}}_{i}=0;\;\;\;{\bar{c}}_{i-1}<\xi <{\bar{c}}_{i}, \end{array} \end{array}$$

(20c) $$\begin{array}{c} \begin{array}{c} \frac{d^{2}{\bar{U}}_{N+1}}{d\xi^{2}}+\frac{\left(1+\chi_{1}^{2}\right)\varpi^{2}}{\left(1+\chi_{2}^{2}\right)+\left(1+\chi_{3}^{2}\right)\left({\bar{H}}_{y}^{2}+{\bar{H}}_{z}^{2}\right)-\mu^{2}\varpi^{2}\left(1+\chi_{1}^{2}\right)}{\bar{U}}_{N+1}=0;\;\;\;{\bar{c}}_{N}<\xi <1 \end{array} \end{array}$$

where i=2,3,…,N, and in view of Equations (17a) and (17b), the dimensionless interfacial conditions read:

(21a) $$\begin{array}{c} \begin{array}{c} {\bar{k}}_{i}\;{\bar{U}}_{i}\left({\bar{c}}_{i}\right)+\left(\left(1+\chi_{2}^{2}\right)+\left(1+\chi_{3}^{2}\right)\left({\bar{H}}_{y}^{2}+{\bar{H}}_{z}^{2}\right)-\mu^{2}\varpi^{2}\left(1+\chi_{1}^{2}\right)\right)\frac{d{\bar{U}}_{i}\left({\bar{c}}_{i}\right)}{d\xi }={\bar{k}}_{i}\;{\bar{U}}_{i+1}\left({\bar{c}}_{i}\right), \end{array} \end{array}$$

(21b) $$\begin{array}{c} \begin{array}{c} \frac{d{\bar{U}}_{i}\left({\bar{c}}_{i}\right)}{d\xi }=\frac{d{\bar{U}}_{i+1}\left({\bar{c}}_{i}\right)}{d\xi };\;i=1,2,\ldots ,N. \end{array} \end{array}$$

For the FIFI and FIFR boundary conditions (see Equations (18a) and (18b)), the following relations should be satisfied by the amplitude functions of the constitutive segments of the defected nanorod at its ends:

(22a) $$\begin{array}{c} \begin{array}{c} \mathrm{FIFI}:\;\;\;\;{\bar{U}}_{1}\left(0\right)=0;\;\;{\bar{U}}_{N+1}\left(1\right)=0, \end{array} \end{array}$$

(22b) $$\begin{array}{c} \begin{array}{c} \mathrm{FIFR}:\;\;\;\;{\bar{U}}_{1}\left(0\right)=0;\;\;\frac{d{\bar{U}}_{N+1}\left(1\right)}{d\xi }=0. \end{array} \end{array}$$

By assuming $\varpi <\sqrt{\frac{\left(1+\chi_{2}^{2}\right)+\left(1+\chi_{3}^{2}\right)\left({\bar{H}}_{y}^{2}+{\bar{H}}_{z}^{2}\right)}{\mu^{2}\left(1+\chi_{1}^{2}\right)}}$, the general solution to Equations (20a) and (20b) could be readily sought as:

(23) $$\begin{array}{c} {\bar{U}}_{k}\left(\xi \right)=A_{k}\cos\left(\Lambda \xi \right)+B_{k}\sin\left(\Lambda \xi \right);\;k=1,2,\ldots ,N+1, \end{array}$$

where $A_{k}$ and $B_{k}$ are the unknown constants, and

(24) $$\begin{array}{c} \Lambda =\frac{\varpi \;\sqrt{1+\chi_{1}^{2}}}{\sqrt{\left(1+\chi_{2}^{2}\right)+\left(1+\chi_{3}^{2}\right)\left({\bar{H}}_{y}^{2}+{\bar{H}}_{z}^{2}\right)-\mu^{2}\varpi^{2}\left(1+\chi_{1}^{2}\right)}}. \end{array}$$

To evaluate these unknowns, the given boundary conditions in Equations (21a), (21b), (22a), and (22b) should be carefully enforced. By introducing Equation (23) to these conditions, the following set of eigenvalue relations would be derivable:

(25) $$\begin{array}{c} Lx=0, \end{array}$$

where

(26) $$\begin{array}{c} x^{T}=<A_{1},B_{1},A_{2},B_{2},\ldots ,A_{i},B_{i},\ldots ,A_{N+1},B_{N+1}>, \end{array}$$

and the nonzero elements of the matrix L for the cases of the FIFR and FIFI boundary conditions are provided in Appendix A. A nontrivial solution to Equation (25) with 2N+2 unknowns exists if and only if detL = 0. By solving this nonlinear relation via bisection approach, all natural frequencies of the nonlocal-surface energetic nanorod with arbitrarily distributed defects could be determined. For the special case of a magnetically affected nanorod with an individual defect, the explicit expressions of the characteristic equations as well as the analytical mode shapes pertinent to the FIFI and FIFR conditions are provided in Appendix B.

## 3. Establishing an NISM for the Magnetically Affected Defected Nanorods

### 3.1. Governing Equations Associated with the NISM

In the context of the integral-based version of the nonlocal continuum theory of Eringen [22], the nonlocal stresses within the solid body (i.e., both BZ and SL) are stated by an integral of the product of a kernel function and the local stresses over the spatial domain. In fact, the kernels are appropriate attenuating functions which are not unique, and for one-, two-, and three-dimensional domains have been introduced by Eringen in his conspicuous book [22]. On the other hand, the applied magnetic forces on both BZ and SL of the defected nanorod are not conservative at all; actually, these influential forces are displacement-dependent and they are incorporated into the longitudinal stiffness of the nanorod. The wholly classical magnetic force applied on both BZ and SL of the defected nanorod is simply displayed by Equation (5), and the resultant classical force within the nanorod accounting for both elastic and magnetic axial rigidities is given by Equation (7).

The explanations mentioned above guide us to express the nonlocal-integral stresses within the SL and BZ of the magnetically affected *i*th nanorod’s segment in terms of the displacement by:

(27a) $$\begin{array}{c} \begin{array}{c} \sigma_{b,i}^{nl}\left(x,t\right)=\int_{\Omega_{i}}\Gamma_{b}\left(\right|x^{*}-x\left|;e_{0}a\right)\;\left(E_{b}+\eta_{b}\left(H_{y}^{2}+H_{z}^{2}\right)\right)\;\frac{\partial u_{i}}{\partial x}\left(x^{*},t\right)\;d\Omega^{*}, \end{array} \end{array}$$

(27b) $$\begin{array}{c} \begin{array}{c} \sigma_{s,i}^{nl}\left(x,t\right)=\int_{S_{i}}\Gamma_{s}\left(\right|x^{*}-x\left|;e_{0}a\right)\;\left(E_{0}+\eta_{0}\left(H_{y}^{2}+H_{z}^{2}\right)\right)\;\frac{\partial u_{i}}{\partial x}\left(x^{*},t\right)\;dS^{*}, \end{array} \end{array}$$

where $\sigma_{b,i}^{nl}$ and $\sigma_{s,i}^{nl}$ denote the nonlocal stresses of the BZ and the SL of the *i*th segment, x is the coordinate of a point from the continuum of the *i*th nanorod, $\Gamma_{b}$ and $\Gamma_{s}$ represent the kernel functions pertinent to the BZ and the SL. Finally, $d\Omega^{*}$ and $dS^{*}$ are the volume and surface of arbitrarily infinitesimal elements from the BZ and the SL, respectively. Let us consider: $\Gamma_{b}=\Gamma_{b0}\;g(|x|;e_{0}a)$ and $\Gamma_{s}=\Gamma_{s0}\;g(|x|;e_{0}a)$, where *g* is an appropriate attenuation function. By assuming $\Gamma_{b0}=\Gamma_{0}/A_{b}$ and $\Gamma_{s0}=\Gamma_{0}/A_{0}$, the requirement of completeness condition for kernel functions yields $\Gamma_{0}={\left[\int_{-\infty }^{\infty }g(|x^{*}\left|;e_{0}a\right)\;dx^{*}\right]}^{-1}$.

Now by taking into account the uniform longitudinal stress in each cross-section, Equations (27a) and (27b) are reduced to:

(28a) $$\begin{array}{c} \begin{array}{c} \sigma_{b,i}^{nl}\left(x,t\right)=\int_{0}^{l_{b}}\Gamma_{b0}\;g(|x^{*}-x\left|;e_{0}a\right)\;\left(E_{b}+\eta_{b}\left(H_{y}^{2}+H_{z}^{2}\right)\right)\;\frac{\partial u_{i}}{\partial x}\left(x^{*},t\right)\;dx^{*}, \end{array} \end{array}$$

(28b) $$\begin{array}{c} \begin{array}{c} \sigma_{s,i}^{nl}\left(x,t\right)=\int_{0}^{l_{b}}\Gamma_{s0}\;g(|x^{*}-x\left|;e_{0}a\right)\;\left(E_{0}+\eta_{0}\left(H_{y}^{2}+H_{z}^{2}\right)\right)\;\frac{\partial u_{i}}{\partial x}\left(x^{*},t\right)\;dx^{*}. \end{array} \end{array}$$

The whole nonlocal-integral-based longitudinal force within the *i*th segment is evaluated by summing up the integration of the given nonlocal stresses in Equations (28a) and (28b) over the cross-sections of the BZ and the SL, respectively. Therefore,

(29) $$\begin{array}{cc} N_{i}^{nl}\left(x,t\right)= & \left[\left(E_{b}A_{b}+E_{0}A_{0}\right)+\left(\eta_{b}A_{b}+\eta_{0}A_{0}\right)\left(H_{y}^{2}+H_{z}^{2}\right)\right]\times \;\\ & \int_{0}^{l_{b}}\Gamma_{0}\;g(|x^{*}-x\left|,e_{0}a\right)\;\frac{\partial u_{i}}{\partial x}\left(x^{*},t\right)\;dx^{*}. \end{array}$$

By introducing Equation (29) to Equation (8), the nonlocal-integral-partial differential equation of the *i*th segment of the magnetically affected nanorod with defects is expressed by:

(30) $$\begin{array}{c} \left(\rho_{b}A_{b}+\rho_{0}A_{0}\right)\frac{\partial^{2}u_{i}}{\partial t^{2}}-\frac{\partial }{\partial x}\;\left\{\begin{array}{c} \left[\left(E_{b}A_{b}+E_{0}A_{0}\right)+\left(\eta_{b}A_{b}+\eta_{0}A_{0}\right)\left(H_{y}^{2}+H_{z}^{2}\right)\right]\times \;\;\\ \int_{0}^{l_{b}}\Gamma_{0}\;g(|x^{*}-x\left|,e_{0}a\right)\;\frac{\partial u_{i}}{\partial x}\left(x^{*},t\right)\;dx^{*} \end{array}\right\}=0, \end{array}$$

with the following interfacial boundary conditions:

(31a) $$\begin{array}{c} \begin{array}{cc} k_{i}\left[u_{i+1}\left(c_{i},t\right)-u_{i}\left(c_{i},t\right)\right]= & \left[\left(E_{b}A_{b}+E_{0}A_{0}\right)+\left(\eta_{b}A_{b}+\eta_{0}A_{0}\right)\left(H_{y}^{2}+H_{z}^{2}\right)\right]\times \;\\ & \int_{c_{i-1}}^{c_{i}}\Gamma_{0}\;g(|x^{*}-c_{i}\left|,e_{0}a\right)\;\frac{\partial u_{i}}{\partial x}\left(x^{*},t\right)\;dx^{*};\;i=1,2,\ldots ,N, \end{array} \end{array}$$

(31b) $$\begin{array}{c} \begin{array}{c} \int_{c_{i-1}}^{c_{i}}\Gamma_{0}\;g(|x^{*}-c_{i}|,e_{0}a)\;\frac{\partial u_{i}}{\partial x}\left(x^{*},t\right)\;dx^{*}=\int_{c_{i}}^{c_{i+1}}\Gamma_{0}\;g(|x^{*}-c_{i}\left|,e_{0}a\right)\;\frac{\partial u_{i+1}}{\partial x}\left(x^{*},t\right)\;dx^{*}, \end{array} \end{array}$$

while the conditions for FIFI and FIFR nanorods are displayed by:

(32a) $$\begin{array}{c} \begin{array}{c} \mathrm{FIFI}:\;\;u_{1}\left(0,t\right)=0,\;\;u_{N+1}\left(l_{b},t\right)=0, \end{array} \end{array}$$

(32b) $$\begin{array}{c} \begin{array}{c} \mathrm{FIFR}:\;\;u_{1}\left(0,t\right)=0,\;\;\int_{c_{N}}^{l_{b}}g(|x^{*}-l_{b}\left|,e_{0}a\right)\;\frac{\partial u_{N+1}}{\partial x}\left(x^{*},t\right)\;dx^{*}=0. \end{array} \end{array}$$

The (*N*+1) nonlocal-integro-partial differential equations in Equation (30) with the provided boundary conditions in Equations (31) and (32) furnish us regarding the equations of motion of the nonlocal-integral-surface energy model with their appropriate conditions. To solve these for natural frequencies, a finite-element-based approach is proposed in the next part.

### 3.2. Frequency Analysis via FEM

Let us rewrite Equation (30) in terms of the nonlocal-integral-based axial forces within the constitutive segments of the magnetically affected-defected nanorod as follows:

(33) $$\begin{array}{c} \left(\rho_{0}A_{0}+\rho_{b}A_{b}\right)\frac{\partial^{2}u_{n}}{\partial t^{2}}-\frac{\partial N_{n}^{nl}}{\partial x}=0;\;n=1,2,\ldots ,N+1, \end{array}$$

where the nonlocal axial forces are displayed by Equation (29). In order to seek harmonic solutions to these relations, the Galerkin method is applied. By pre-multiplying both sides of Equation (33) by the variation of the displacement field of the *i*th segment, namely $\delta u_{n}\left(x,t\right)$, and by integrating the resulted statement over the length of the *n*th segment, one can write:

(34) $$\begin{array}{c} \sum_{n=1}^{N+1}\left\{\int_{c_{n-1}}^{c_{n}}\;\left[\left(\rho_{b}A_{b}+\rho_{0}A_{0}\right)\;\delta u_{n}\;\frac{\partial^{2}u_{n}}{\partial t^{2}}+\frac{\partial \delta u_{n}}{\partial x}\;N_{n}^{nl}\right]dx-{\left[\delta u_{n}N_{n}^{nl}\right]}_{c_{m-1}}^{c_{m}}\right\}=0. \end{array}$$

The longitudinal displacements of the constitutive segments (i.e., $u_{n}$; *n* = 1, 2, …, *N*+1) as well as their variations can be discretized in terms of the FEM shape functions as: $u_{n}\left(x,t\right)=\sum_{i=1}^{NE^{n}}U_{i}^{n}\left(t\right)\;N_{i}^{n}\left(x\right)$, in which $N_{i}^{n}\left(x\right)$ is the vector of shape functions associated with the *i*th element of the *m*th segment (for example, it is a column vector whose size would be 2 for two-nodes-based elements), $U_{i}^{n}\left(t\right)$ denotes the unknown time-varying vector, and $NE^{n}$ is the number of elements used for discretization of the longitudinal displacement field of the *n*th segment. By introducing the recent relation to Equation (34) through imposing the given boundary conditions in Equations (31) and (32), one can arrive at:

(35) $$\begin{array}{c} M\frac{d^{2}U}{dt^{2}}+K\;U=0, \end{array}$$

where the vector of the unknown parameters as well as the mass and stiffness matrices are defined by:

(36a) $$\begin{array}{c} \begin{array}{c} U\left(t\right)={\left\{U_{1}^{1}\left(t\right),U_{2}^{1}\left(t\right),\ldots ,U_{N_{e}^{1}}^{1}\left(t\right),\ldots ,U_{1}^{N+1}\left(t\right),U_{2}^{N+1}\left(t\right),\ldots ,U_{N_{e}^{N+1}}^{N+1}\left(t\right)\right\}}^{T}, \end{array} \end{array}$$

(36b) $$\begin{array}{c} \begin{array}{c} M=\sum_{n=1}^{N+1}\int_{c_{n-1}}^{c_{n}}\left(\rho_{b}A_{b}+\rho_{0}A_{0}\right)N_{i}^{n}\left(x\right){\left(N_{j}^{n}\right)}^{T}\left(x\right)\;dx, \end{array} \end{array}$$

(36c) $$\begin{array}{c} \begin{array}{cc} K= & \sum_{n=1}^{N+1}\int_{c_{n-1}}^{c_{n}}\int_{c_{n-1}}^{c_{n}}\left\{\begin{array}{c} \left[\left(E_{b}A_{b}+E_{0}A_{0}\right)+\left(\eta_{b}A_{b}+\eta_{0}A_{0}\right)\left(H_{y}^{2}+H_{z}^{2}\right)\right]\times \;\\ \Gamma_{0}\;g(|x^{*}-x\left|,e_{0}a\right)\;\frac{dN_{i}^{n}}{dx}\left(x\right)\frac{d{\left(N_{j}^{n}\right)}^{T}}{dx}\left(x^{*}\right) \end{array}\right\}\;dx^{*}dx\;\\ & +\sum_{n=1}^{N}\;k_{n}\left(N_{i}^{n+1}\left(c_{n}\right)-N_{i}^{n}\left(c_{n}\right)\right)\left({\left(N_{j}^{n+1}\right)}^{T}\left(c_{n}\right)-{\left(N_{j}^{n}\right)}^{T}\left(c_{n}\right)\right)\;\\ & +\sum_{n=1}^{2}\;\alpha_{p_{n}}\left(N_{i}^{1}\left(x_{n}\right){\left(N_{j}^{1}\right)}^{T}\left(x_{n}\right)+N_{i}^{N+1}\left(x_{n}\right){\left(N_{j}^{N+1}\right)}^{T}\left(x_{n}\right)\right), \end{array} \end{array}$$

where $x_{1}=0$, $x_{2}=l_{b}$, and $\alpha_{p_{m}}$ are the penalty factors used for enforcing the essential boundary conditions. For the FIFR and FIFI end conditions, we set ($\alpha_{p_{1}}$,$\alpha_{p_{2}}$) = $10^{8}\;E_{b}A_{b}$(1,0) and $10^{8}\;E_{b}A_{b}$(1,1), respectively.

To investigate the free vibration of the magnetically affected nanorod with multiple defects more conveniently, we use effectively the dimensionless quantities given in Equation (15). As a result, the dimensionless set of equations takes the following form:

(37) $$\begin{array}{c} \bar{M}\frac{d^{2}\bar{U}}{d\tau^{2}}+\bar{K}\;\bar{U}=0, \end{array}$$

where

(38a) $$\begin{array}{c} \begin{array}{c} \bar{U}\left(\tau \right)={\left\{{\bar{U}}_{1}^{1}\left(\tau \right),{\bar{U}}_{2}^{1}\left(\tau \right),\ldots ,{\bar{U}}_{N_{e}^{1}}^{1}\left(\tau \right),\ldots ,{\bar{U}}_{1}^{N+1}\left(\tau \right),{\bar{U}}_{2}^{N+1}\left(\tau \right),\ldots ,{\bar{U}}_{N_{e}^{N+1}}^{N+1}\left(\tau \right)\right\}}^{T}, \end{array} \end{array}$$

(38b) $$\begin{array}{c} \begin{array}{c} \bar{M}=\sum_{n=1}^{N+1}\int_{{\bar{c}}_{n-1}}^{{\bar{c}}_{n}}\left(1+\chi_{1}^{2}\right)N_{i}^{n}\left(\xi \right){\left(N_{j}^{n}\right)}^{T}\left(\xi \right)\;d\xi , \end{array} \end{array}$$

(38c) $$\begin{array}{c} \begin{array}{cc} \bar{K}= & \sum_{n=1}^{N+1}\int_{{\bar{c}}_{n-1}}^{{\bar{c}}_{n}}\int_{{\bar{c}}_{n-1}}^{{\bar{c}}_{n}}\left\{\begin{array}{c} \left[\left(1+\chi_{2}^{2}\right)+\left(1+\chi_{3}^{2}\right)\left({\bar{H}}_{y}^{2}+{\bar{H}}_{z}^{2}\right)\right]\times \;\\ {\bar{\Gamma }}_{0}\;g(|\xi^{*}-\xi \left|,\mu \right)\;\frac{dN_{i}^{n}}{d\xi }\left(\xi \right)\frac{d{\left(N_{j}^{n}\right)}^{T}}{d\xi }\left(\xi^{*}\right) \end{array}\right\}\;d\xi d\xi^{*}\;\\ & +\sum_{n=1}^{N}\;{\bar{k}}_{n}\left(N_{i}^{n+1}\left({\bar{c}}_{n}\right)-N_{i}^{n}\left({\bar{c}}_{n}\right)\right)\left({\left(N_{j}^{n+1}\right)}^{T}\left({\bar{c}}_{n}\right)-{\left(N_{j}^{n}\right)}^{T}\left({\bar{c}}_{n}\right)\right)\;\\ & +\sum_{n=1}^{2}\;{\bar{\alpha }}_{p_{n}}\left(N_{i}^{1}\left(\xi_{n}\right){\left(N_{j}^{1}\right)}^{T}\left(\xi_{n}\right)+N_{i}^{N+1}\left(\xi_{n}\right){\left(N_{j}^{N+1}\right)}^{T}\left(\xi_{n}\right)\right), \end{array} \end{array}$$

and

(39) $$\begin{array}{c} \xi_{1}=0,\;\;\xi_{2}=1,\;\;{\bar{U}}_{i}^{j}=\frac{U_{i}^{j}}{l_{b}},\;\;\;{\bar{\Gamma }}_{0}=\Gamma_{0}l_{b},\;\;\;{\bar{\alpha }}_{p_{m}}=\frac{\alpha_{p_{m}}}{E_{b}A_{b}}. \end{array}$$

## 4. Results and Discussion

Take into account a nanorod made from silver with the following mechanical properties: *E${}_{b}$*=76 GPa, $\rho_{b}$ = 10,500 kg/m${}^{3}$, $\rho_{0}$ = $10^{-7}$ kg/m${}^{2}$, *E${}_{0}$* = 1.22 N/m, and $\tau_{0}$ = 0.89 N/m. Concerning the magnetic permeability of such nanorods, the value of this factor for the nanorod’s bulk of silver is rationally considered to be equal to that of the free space, namely $\eta_{b}$ = $1.256637\times 10^{-7}$ N/A${}^{2}$. Furthermore, the magnetic permeability of the SL is assumed to be $1.256637\times 10^{-17}$ N.m/A${}^{2}$. In the following, we provide a comparison study, and then, the roles of influential factors on the free vibration behavior of defected nanorods subjected to transverse magnetic fields are explained and discussed in some details.

### 4.1. Several Comparison Studies

#### 4.1.1. A Particular Verification Study

In order to become ensure on some parts of the performed calculations, we compare the predicted free dynamic response by the proposed model with those of Hsu et al. [82] for the case of the magnetic field free. These researchers studied longitudinal vibrations of nanorods by developing a nonlocal model. Herein we established a more sophisticated model accounting for both nonlocality and surface energy effect as well. In the lack of the surface and nonlocal effects, the predicted results by the suggested model and those of Hsu et al. [82] have been presented in Table 1 for FIFI-based and FIFR-based damaged nanorods. As it is seen, there exists a good agreement between the estimated first six frequencies of the defected nanostructure and those of Hsu et al. [82].

#### 4.1.2. NDSM vs. NISM

To check the proposed NISM using FEM, its natural frequencies are verified with those of NDSM using the suggested semi-analytical solution. For the NISM based on the FEM, each segment is divided into 20 identical elements with two nodes at the ends and Hermitian shape functions, the attenuation function is considered to be: $g\left(x;e_{0}a\right)=\exp(-|x|/\left(e_{0}a\right))$, and the essential boundary conditions are imposed by the penalty method as explained in Section 3.2.

In Table 2 and Table 3, the predicted first two longitudinal frequencies of the defected nanorod under the transverse magnetic field with ${\bar{H}}_{y}$ = ${\bar{H}}_{z}$ = 1 based on the NDSM and NISM have been presented for the FIFI and FIFR ends, respectively. The results are provided for four numbers of defects (i.e., *N* = 2, 3, 4, and 5) and four nanorod lengths (i.e., $l_{b}$ = 10, 15, 20, 25, and 30 nm). In most of the cases, the predicted frequencies by the NISM are close to those of the NDSM. A careful scrutiny of the obtained results for the FIFI-defected nanorod reveals that the relative differences between the predicted fundamental frequencies by the NDSM and those estimated based on the NISM would generally reduce by lessening the nanorod’s length or increasing the number of defects. Such a trend is not exactly right for the predicted second frequencies. Concerning the magnetically affected nanorod with FIFI ends, these maximum relative differences for the first and second frequencies are approximately equal to 8.8 and 5.7 percent, which are observed in the cases of (*N*,$l_{b}$) = (2,30) and (3,30), respectively. Regarding the magnetically affected nanorod with FIFR ends, the maximum differences between the NISM’s results and the NDSM’s results in order are reported to be 20.2 and 6.7 percent for the first and the second frequencies, in the case of (*N*,$l_{b}$) = (6,30). In other cases, the predicted results by the NISM are very close to those of the NDSM such that the maximum relative differences are lower than 6 percent.

Table 4 and Table 5 display the predicted first three longitudinal frequencies by the NDSM and NISM for magnetically affected nanorods with the FIFI and FIFR ends, respectively. The results are given for four defect parameters (i.e., $k_{i}$ = 2, 3, 4, and 5) and four magnetic field strength values (i.e., ${\bar{H}}_{y}$ = ${\bar{H}}_{z}$ = 1, 2, 3, and 4). Generally, the results of the NDSM are so close to those of the NISM such that the maximum relative differences of the first, second, and third frequencies are lower than 10.5(7.2), 9.36(8.2), and 12.9(7.5) percent for the FIFI(FIFR) defected nanorod. For both FIFR and FIFI end conditions, the above-mentioned relative differences would increase by growing of the defect parameter; however, an increase of the magnetic field strength leads to the lessening of the relative differences between the NDSM’s results and the NISM’s results. A more detailed influence of the transverse magnetic field on the free vibration of the defected nanorod will be explained and discussed in an upcoming subsection.

### 4.2. Effect of the Nonlocality

**Figure 2:**
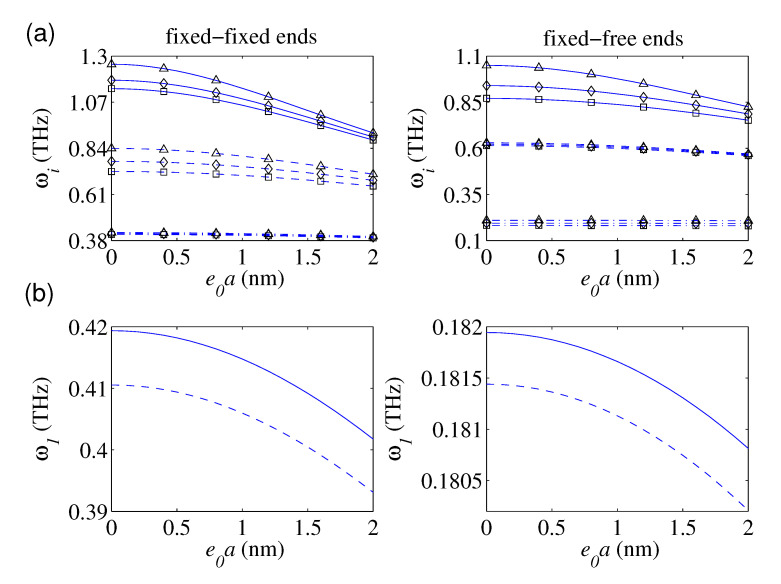
The first three frequencies as a function of $e_{0}a$ for: (**a**) different defect parameters: ($\chi_{1}^{2},\chi_{2}^{2}$ = 0, $c_{1}$ = 8 nm; (−.−) $\omega_{1}$, (−−) $\omega_{2}$, (—) $\omega_{3}$; (□) ${\bar{k}}_{1}$ = 4, (◇) ${\bar{k}}_{1}$ = 8, (Δ) ${\bar{k}}_{1}$ = 10,000), (**b**) both nonlocal elasticity theory (NET)-based and nonlocal surface elasticity theory (NSET)-based models; ($l_{b}$ = 20 nm, $D_{0}$ = 0.5 nm, $c_{1}$ = 8 nm, ${\bar{k}}_{1}$ = 4, *N* = 1, ${\bar{H}}_{y}$ = ${\bar{H}}_{z}$ = 0.1; (−−) NET, (—) NSET).

In Figure 2a, the plots of the first three frequencies of the magnetically affected nanobars with defects under FIFI and FIFR boundary conditions as a function of the nonlocal parameter have been demonstrated. The main aim of these plots is to show how the nonlocality influences on the softening behavior of the defected nanostructure for three damage level of the single defect (i.e., ${\bar{k}}_{1}$ = 4, 8, and 10,000) in the lack of the surface effect (i.e., $\chi_{1}$ = $\chi_{2}$ = 0). By increasing the stiffness of the local defect, the natural frequencies would increase. This fact is more obvious for higher frequencies. Additionally, the variation of the defect parameter is more influential on the nanorod’s frequencies with lower nonlocality. This fact is mainly related to reducing the nanorod’s axial stiffness due to an increase in $e_{0}a$. Figure 2b displays the influence of the $e_{0}a$ on the fundamental frequencies based on the NET and the NSET. According to the plotted results, the predicted fundamental frequencies by the NSET are greater than those of the NET, irrespective of the considered $e_{0}a$. This fact is ascertained to the positive incorporation of the surface axial rigidity into the elastic strain energy of the defected nanobars.

### 4.3. Effect of the Defect’s Location

**Figure 3:**
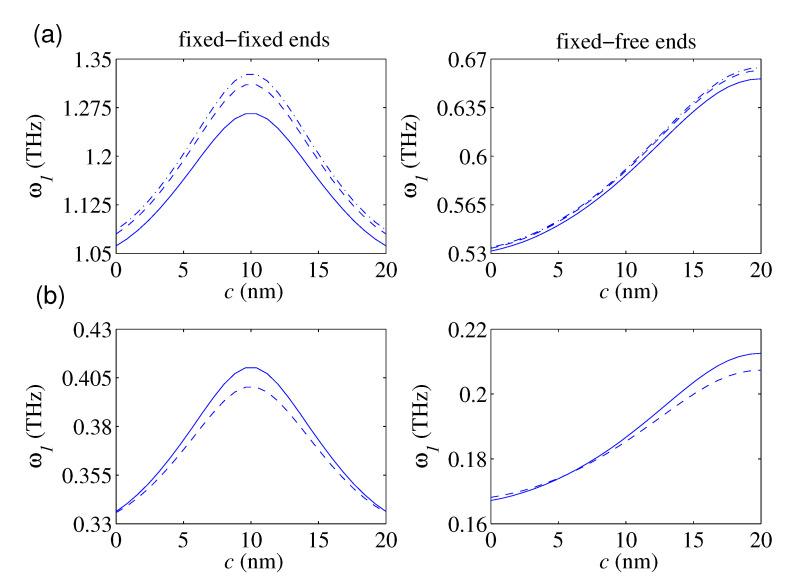
The fundamental frequency in terms of the single defect’s location for: (**a**) three values of $e_{0}a$; ((−.−) $e_{0}a$ = 0, (−−) $e_{0}a$ = 1 nm, (—) $e_{0}a$ = 2 nm; ${\bar{k}}_{1}$ = 4, $\chi_{1}^{2},\chi_{2}^{2}$ = 0), (**b**) both NET-based and NSET-based models; ($l_{b}$ = 20 nm, $D_{0}$ = 0.5 nm, $e_{0}a$ = 2 nm, ${\bar{k}}_{1}$ = 4, *N*=1, ${\bar{H}}_{y}$ = ${\bar{H}}_{z}$ = 0.1; (−−) NET, (—) NSET).

We are also interested in examining the role of the location of the local defect on the free vibration behavior of the defected nanobars. In doing so, in Figure 3a, for a constant value of the defect’s axial stiffness, the plots of the fundamental frequency as a function of defect’s location have been graphed for three nonlocal factors (i.e., *e${}_{0}$a* = 0, 1, and 2 nm) in the lack of surface energy. As seen, the nanostructure’s fundamental frequency enlarges as the defect approach to the center of the FIFI nanorod. For FIFR nanorods, the fundamental frequency would lessen by approaching to its free end. As a general conclusion, as the defect becomes far away from the nanorod’s supports, the nanorod becomes stiffer, and its natural frequencies would grow. This fact is because the elastic strain energy of the defected nanorod with a defect near the support is lesser than that whose defect is father. This issue is valid for each value of $e_{0}a$. Furthermore, irrespective of the defect’s location, the fundamental frequency of FIFI and FIFR nanobars lessens by increasing the nonlocality. Such a fact is more obvious for defected nanorods whose defects are farther from the support. In Figure 3b, the predicted fundamental frequencies of the defected nanorod with FIFI and FIFR ends based on the SET and the NSET have been demonstrated as a function of the defect’s location. The plotted results clearly display that the surface energy’s role on the free vibration behavior of the FIFI nanorods reaches its maximum when the nanorod becomes defected at its midspan point. By approaching the defect to the nanorods supports, the relative discrepancies between the results of the NSET and those of the NET would reduce, and the influence of the surface energy on the vibrational response of the FIFI nanorod lessens. This fact also holds valid for nanorods with FIFR ends such that the maximum influence of the surface energy on the free vibration behavior of the nanostructure is observed when the defect occurs close to the free end. Additionally, in the case of the FIFI end conditions, the predicted natural frequencies by the NSET are greater than those of the NET for all locations of the local defect. However, in the case of the FIFR ends when the defect is fairly close to the fixed-end (i.e., *c* < 5 nm), the estimated fundamental frequency by the NET is greater than that obtained by the NSET. For *c* > 5 nm, the results of the NSET are commonly greater than those of the NET and their discrepancies reach to their maximum level when the defect approaches to the free end.

### 4.4. Effect of the Nanorod’s Diameter

**Figure 4:**
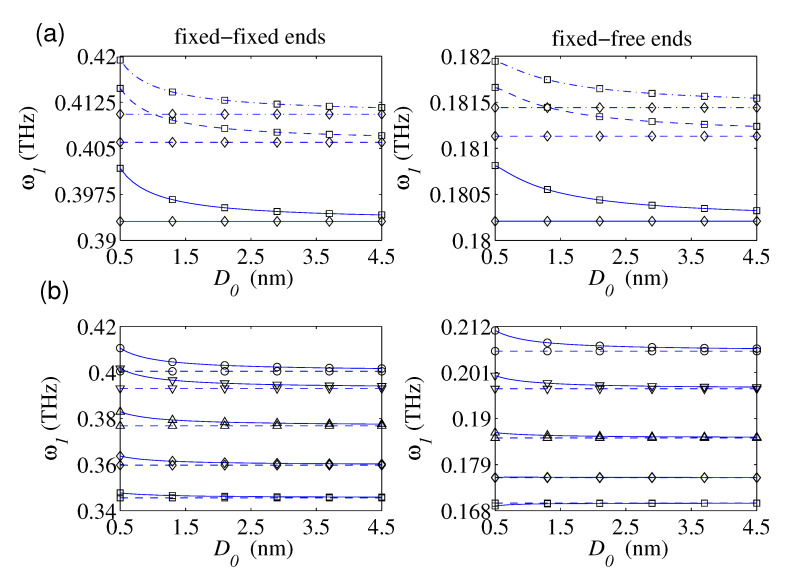
The fundamental frequency as a function of the defected nanorod’s diameter with and without considering the surface effect for: (**a**) different values of $e_{0}a$; ((−.−) $e_{0}a$ = 0, (−−) $e_{0}a$ = 1 nm, (—) $e_{0}a$ = 2 nm; $c_{1}$ = 8 nm, ${\bar{k}}_{1}$ = 4; (◇) $\chi_{1}^{2},\chi_{2}^{2}$ = 0; (□) $\chi_{1}^{2},\chi_{2}^{2}\neq$0), (**b**) different locations of the single defect; ($l_{b}$ = 20 nm, $e_{0}a$ = 2 nm, ${\bar{k}}_{1}$ = 4, *N* = 1, ${\bar{H}}_{y}$ = ${\bar{H}}_{z}$ = 0.1; (□) $c_{1}$ = 2, (Δ) $c_{1}$ = 6, (∇) $c_{1}$ = 8 nm, (∘) $c_{1}$ = 10 nm; (−−) NET, (—) NSET).

For three small-scale parameters (i.e., *e${}_{0}$ a* = 0, 1, and 2 nm), the graphs of the fundamental frequency in terms of the defected nanorod’s diameter have been provided in Figure 4a for the cases of the FIFI and FIFR conditions. The predicted results are based on the NET and the NSET in the case of $c_{1}$ = 8 nm and ${\bar{k}}_{1}$ = 4. For a given small-scale parameter, the NET displays that the variation of the diameter of the defected nanorod does not influence on the variation of its fundamental frequency. However, the plotted results based on the NSET obviously show that the fundamental frequency would decrease by reducing the nanorod’s diameter. Factually, the ratio of the surface to the volume of the defected nanorod would reduce as its diameter increases, and thereby, the effect of the surface energy on the free vibration behavior of the nanostructure diminishes by growing the nanorod’s diameter. This important issue could not be displayed by the suggested model by Hsu et al. [82]. Figure 4b demonstrates variation of the fundamental frequencies of both FIFI and FIFR defected nanorods as a function of the diameter for several locations of the defect based on the NET and the NSET. As it is seen, the relative discrepancies between the results of the NSET and those of the NET would increase as the nanorod’s becomes thinner and the distance of the defect from the support increases. It implies that the need for consideration of the surface energy intensifies as the defect approaches to the support of the nanorod. Furthermore, for all considered locations of the defect, the predicted results by the NSET would approach to those of the NET as the diameter of the locally defected nanorod increases.

### 4.5. Effect of the Magnetic Field Strength

**Figure 5:**
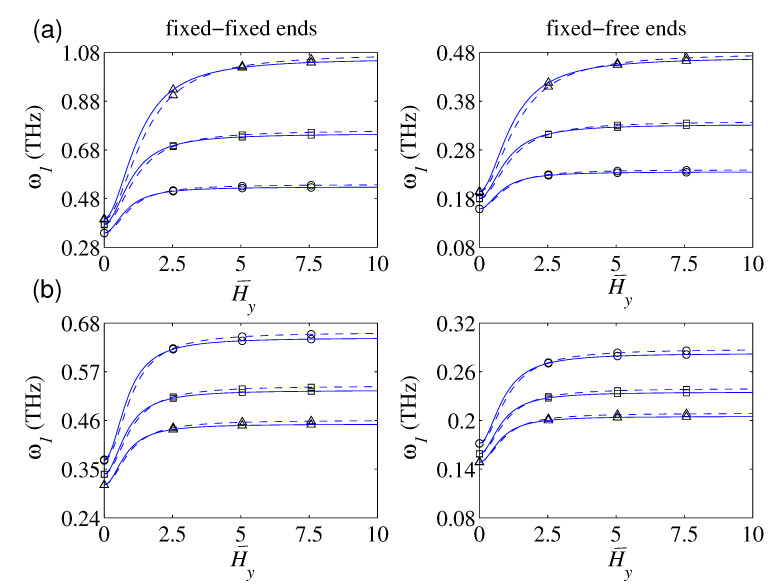
The fundamental frequency in terms of the magnetic field strength with and without considering the surface effect: (**a**) different defect parameters; ((∘) ${\bar{k}}_{1}$ = 4, (□) ${\bar{k}}_{1}$ = 8, (Δ) $k_{1}$ = 16; *N* = 3), (**b**) different number of defects; ((∘) *N* = 2, (□) *N* = 3, (Δ) *N* = 4; $k_{i}$ = 4); ($e_{0}a$ = 1 nm, $l_{b}$ = 20 nm, $D_{0}$ = 1 nm; (−−) NET, (—) NSET).

The role of the magnetic field strength on the free vibration of the defected nanorod is investigated in this part. Figure 5a shows fundamental frequencies of the FIFI and FIFR nanorods vs. magnetic field strength. The derived results are graphed in the case of $H_{y}$ = $H_{z}$, three uniform defects (i.e., ${\bar{c}}_{i}$ = $\frac{i}{4}$), and for three defect factors (i.e., $k_{1}$ = 4, 8, and 16). According to the presented results, two distinct branches are detectable. In the first branch, the predicted fundamental frequency by both NET and NSET grows by growing the magnetic field strength. Further, the influence of the defect parameter on the plots’ growth is so apparent; actually, the variation of the magnetic field strength on the variation of the fundamental frequency of defected nanorods with a higher defect factor is more noticeable. For the magnetic field strength greater than a particular value, the fundamental frequency of the defected nanorod trivially varies by enlarging the magnetic field strength. This is the main characteristic of the second branch, and the aforementioned particular value strongly relies on the defect factor. The demonstrated results indicate that a higher defect factor results in a higher particular value.

Figure 5b shows the plots of fundamental frequencies in terms of the magnetic field strength for three configurations of the uniform defects (i.e., *N* = 3, 4, and 5) in the case of $k_{i}$ = 4. Irrespective of the applied magnetic field, the frequency reduces by increasing the number of defects. In the second branch (i.e., a magnetic field greater than a specific value), such a reduction rate is slightly affected by the strength of the magnetic field. However, in the first branch, the amount of reduction in fundamental frequency due to an increase in the number of defects depends on the magnetic field strength.

### 4.6. Effect of the Number of Defects

**Figure 6:**
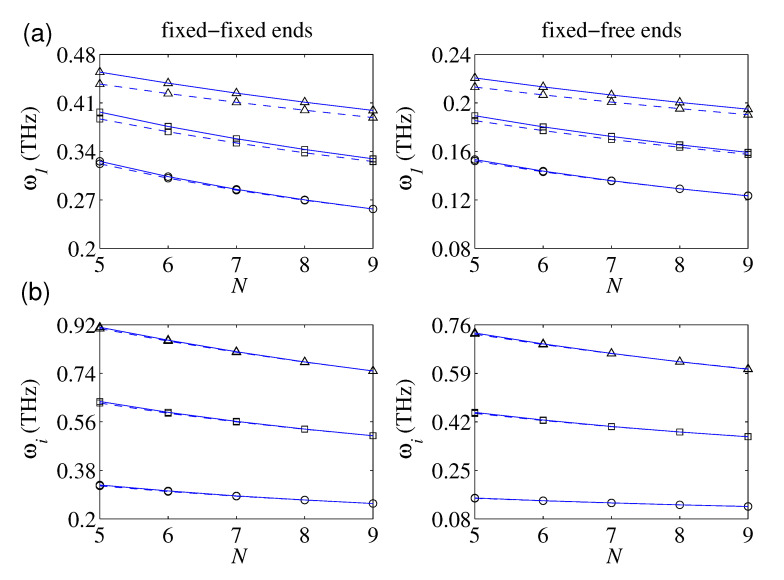
The natural frequencies as a function of the number of uniformly placed defects with and without considering the surface effect: (**a**) fundamental frequency for different defect parameters; ((∘) ${\bar{k}}_{i}$ = 4, (□) ${\bar{k}}_{i}$ = 8, (Δ) $k_{i}$ = 16) (**b**) first, second, and third frequencies for the case of ${\bar{k}}_{i}$ = 4; ((∘) $\omega_{1}$, (□) $\omega_{2}$, (Δ) $\omega_{3}$; $l_{b}$ = 20 nm, $D_{0}$ = 1 nm, $e_{0}a$ = 1 nm, ${\bar{c}}_{i}\;=\;\frac{i}{N+1}$, ${\bar{H}}_{y}$ = ${\bar{H}}_{z}$ = 0.5; (−−) NET, (—) NSET).

The influence of the number of local defects on the vibration behavior of the magnetically affected nanorod is of great interest. In Figure 6a, the plotted results of the fundamental frequency in terms of the number of uniformly caused defects (i.e., ${\bar{c}}_{i}=\frac{i}{N+1};\;i=1,2,\ldots ,N$ along the nanorod for three defect factors (i.e., ${\bar{k}}_{i}$ = 4, 8, and 16) are presented. As it is seen, the fundamental frequency of the defected nanorod decreases by an increase in the number of local defects for all considered levels of the defect factors. For a given number of defects, the fundamental frequency grows by increasing the defect factor. Additionally, the plotted results based on the NSET are higher than those demonstrated based on the NET for most cases. By increasing the number of uniformly caused defects, the slopes of the plots reduce, irrespective of the defect factor. Actually, the variation of the defects’ number for a lower number of defects is more influential on the axial vibration of the magnetically affected nanorod. It is anticipated that the fundamental frequency of the defected nanostructure approaches to a constant whose value is chiefly influenced by the defect factor and magnetic field strength. Such a fact holds correct for both FIFI and FIFR defected nanorods. This issue guides us that the transverse magnetic field could be effectively employed for controlling axial vibration of fully defected nanorods.

Figure 6b displays the influence of the number of defects on the first three dominant natural frequencies of the magnetically affected defected nanorod for both FIFI and FIFR boundary conditions. The considered frequencies generally reduce as the number of defects reduces. The rate of reduction is more obvious for higher vibrational frequencies.

## 5. Conclusions

Longitudinal vibrations of FIFI and FIFR defected nanorods in the presence of transverse magnetic field were investigated using the nonlocal-differential/integral continuum-based theory of Eringen and the surface elasticity theory of Gurtin–Murdoch. By considering a linear spring model for the locally defected zones of the nanorod, the nanostructure was divided into appropriate segments. By enforcing the appropriate nonlocal-surface energy-based conditions at the interfaces as well as the ends, the explicit dispersion relations for locally defected nanorods with FIFI and FIFR could be derived. In the case of a single defect, their corresponding mode shapes were displayed as well, and in the absence of both magnetic field and surface energy, the results of the nonlocal-differential-based model were successfully verified with those of other research work. Additionally, the nonlocal-integral equations of motion of the magnetically affected rod-like nanostructure were displayed and solved by employing the finite-element method. For a particular kernel function, the predicted results by the nonlocal-integral formulations are compared with those of the nonlocal-differential model. The roles of the nonlocality, stiffness, and location of the local defects, number of defects, surface energy, diameter, and magnetic field strength on the natural frequencies of the defected nanorods were explained in some detail.

One of the crucial findings of this work is to reveal the shortcoming of the nonlocal-differential-based formulations in capturing the free vibration behavior of the locally defected nanorods based on the nonlocal-integral formulations. Another major finding is that, beyond the nonlocality, the surface effect should also be considered in the modeling of the axial vibration of defected nanorod structures. This matter becomes more crucial as the diameter of the defected nanorod or the distance between the defect and the support decreases.

## Figures and Tables

**Table 1:** Verification of the predicted first six dimensionless frequencies by the proposed nonlocal-differential-based model and those obtained by the model of Hsu et al. [82] for both fixed–fixed (FIFI) and fixed–free (FIFR) ends ($l_{b}$ = 20 nm, $c_{1}$ = 4.004 nm, ${\bar{k}}_{1}$ = 8.7413, $e_{0}a$ = 0, $\chi_{1}^{2},\chi_{2}^{2}$ = 0, $H_{y}$ = $H_{z}$ = 0).

Conditions	*i*	1	2	3	4	5	6
FIFI	$\varpi_{i}$ (PS ${}^{§}$)	2.9429	6.2236	9.3013	11.5211	14.5149	18.1339
	$\varpi_{i}$ [82]	2.9429	6.2236	9.3013	11.5211	14.5149	18.1339
FIFR	$\varpi_{i}$ (PS)	1.4278	4.5578	7.8540	10.4472	12.8743	16.2952
	$\varpi_{i}$ [82]	1.4278	4.5576	7.8540	10.4486	12.8741	16.2952

${}^{§}$: PS stands for the present work.

**Table 2:** A verification between the predicted first two longitudinal frequencies of the magnetically affected nanorod with FIFI ends according to the nonlocal-differential-surface energy-based model (NDSM) and those of the nonlocal-integral-surface energy-based model (NISM) (${\bar{k}}_{i}$ = 3, $D_{0}$ = 4 nm, $e_{0}a$ = 1 nm, ${\bar{H}}_{y}$ = ${\bar{H}}_{z}$ = 1).

$l_{b}$	*N* = 2		*N* = 3		*N* = 4		*N* = 5	
(nm)	NDSM	NISM	NDSM	NISM	NDSM	NISM	NDSM	NISM
10	0.9477	1.0204	0.8024	0.8521	0.7073	0.7448	0.6388	0.6683
	2.2301	2.0107	1.4714	1.5521	1.3404	1.4083	1.2307	1.2875
15	0.6341	0.6888	0.5360	0.5736	0.4721	0.5005	0.4262	0.4486
	1.5722	1.4625	0.9856	1.0425	0.8971	0.9458	0.8229	0.8637
20	0.4762	0.5196	0.4023	0.4323	0.3543	0.3769	0.3198	0.3377
	1.2042	1.1430	0.7404	0.7840	0.6737	0.7115	0.6177	0.6495
25	0.3812	0.4170	0.3220	0.3469	0.2835	0.3023	0.2559	0.2708
	0.9730	0.9363	0.5928	0.6281	0.5393	0.5702	0.4944	0.5205
30	0.3178	0.3482	0.2683	0.2896	0.2362	0.2524	0.2132	0.2260
	0.8153	0.7923	0.4941	0.5238	0.4495	0.4757	0.4121	0.4342

**Table 3:** A verification between the predicted first two longitudinal frequencies of the magnetically affected nanorod with FIFR ends according to the NDSM and those of the NISM (${\bar{k}}_{i}$ = 3, $D_{0}$ = 4 nm, $e_{0}a$ = 1 nm, ${\bar{H}}_{y}$ = ${\bar{H}}_{z}$ = 1).

$l_{b}$	*N* = 2		*N* = 3		*N* = 4		*N* = 5	
(nm)	NDSM	NISM	NDSM	NISM	NDSM	NISM	NDSM	NISM
10	0.4173	0.4287	0.3631	0.3756	0.3254	0.3367	0.2974	0.3071
	1.1600	1.2215	1.0413	1.0886	0.9473	0.9867	0.8731	0.9064
15	0.2784	0.2898	0.2421	0.2533	0.2170	0.2267	0.1983	0.2065
	0.7762	0.8216	0.6960	0.7315	0.6327	0.6621	0.5829	0.6077
20	0.2088	0.2189	0.1816	0.1910	0.1628	0.1708	0.1487	0.1555
	0.5828	0.6184	0.5225	0.5507	0.4748	0.4982	0.4374	0.4571
25	0.1671	0.1758	0.1453	0.1532	0.1302	0.1372	0.1190	0.1235
	0.4665	0.4957	0.4181	0.4415	0.3800	0.3994	0.3500	0.3659
30	0.1393	0.1468	0.1211	0.1246	0.1085	0.1025	0.0992	0.1243
	0.3889	0.4136	0.3485	0.3676	0.3167	0.3303	0.2917	0.3127

**Table 4:** A verification between the predicted first three longitudinal frequencies of the magnetically affected nanorod with FIFI ends according to the NDSM and those of the NISM ($l_{b}$ = 20 nm, *N* = 3, $D_{0}$ = 4 nm, $e_{0}a$ = 1 nm).

${\bar{H}}_{y}$	${\bar{k}}_{i}$ = 2		${\bar{k}}_{i}$ = 3		${\bar{k}}_{i}$ = 4		${\bar{k}}_{i}$ = 5	
	NDSM	NISM	NDSM	NISM	NDSM	NISM	NDSM	NISM
1	0.3434	0.3610	0.4023	0.4323	0.4456	0.4886	0.4790	0.5354
	0.6216	0.6453	0.7404	0.7840	0.8311	0.8981	0.9031	0.9963
	1.5239	1.3700	1.5609	1.4172	1.5947	1.4633	1.6253	1.5082
2	0.3663	0.3726	0.4414	0.4528	0.5016	0.5188	0.5522	0.5756
	0.6447	0.6523	0.7827	0.7968	0.8959	0.9176	0.9927	1.0232
	2.5658	2.2804	2.5903	2.3081	2.6141	2.3358	2.6374	2.3633
3	0.3727	0.3758	0.4529	0.4585	0.5189	0.5275	0.5758	0.5875
	0.6506	0.6542	0.7936	0.8002	0.9127	0.9228	1.0162	1.0305
	3.6977	3.2780	3.7151	3.2971	3.7322	3.3162	3.7492	3.3353
4	0.3752	0.3770	0.4575	0.4607	0.5259	0.5309	0.5853	0.5922
	0.6529	0.6549	0.7977	0.8015	0.9190	0.9248	1.0251	1.0332
	4.8577	4.3023	4.8710	4.3168	4.8842	4.3313	4.8974	4.3458

**Table 5:** A verification between the predicted first three longitudinal frequencies of the magnetically affected nanorod with FIFR ends according to the NDSM and those of the NISM ($l_{b}$ = 20 nm, *N* = 3, $D_{0}$ = 4 nm, $e_{0}a$ = 1 nm).

${\bar{H}}_{y}$	${\bar{k}}_{i}$ = 2		${\bar{k}}_{i}$ = 3		${\bar{k}}_{i}$ = 4		${\bar{k}}_{i}$ = 5	
	NDSM	NISM	NDSM	NISM	NDSM	NISM	NDSM	NISM
1	0.1542	0.1599	0.1816	0.1910	0.2022	0.2153	0.2184	0.2352
	0.4407	0.4567	0.5225	0.5507	0.5846	0.6265	0.6343	0.6908
	0.6552	0.6752	0.7860	0.8227	0.8888	0.9451	0.9731	1.0512
2	0.1634	0.1656	0.1972	0.2011	0.2245	0.2302	0.2476	0.2552
	0.4614	0.4669	0.5587	0.5687	0.6380	0.6531	0.7054	0.7263
	0.6735	0.6800	0.8194	0.8314	0.9398	0.9583	1.0438	1.0696
3	0.1661	0.1671	0.2019	0.2038	0.2315	0.2344	0.2571	0.2610
	0.4671	0.4697	0.5689	0.5737	0.6534	0.6608	0.7266	0.7368
	0.6782	0.6812	0.8280	0.8336	0.9531	0.9618	1.0623	1.0745
4	0.1671	0.1677	0.2038	0.2049	0.2344	0.2361	0.2609	0.2633
	0.4692	0.4708	0.5729	0.5757	0.6595	0.6637	0.7350	0.7409
	0.6799	0.6817	0.8313	0.8345	0.9582	0.9632	1.0694	1.0763

## Appendix A. The Elements of the L Matrix

For the case of the FIFI condition, the nonzero elements of the matrix L are as,

(A1) $$\begin{array}{c} L_{11}=1,\;\;L_{2(2N+1)}=\cos\left(\Lambda \right),\;\;L_{2(2N+2)}=\sin\left(\Lambda \right),\;\\ L_{\left(2i+1)(2i-1\right)}=-\sin\left(\Lambda {\bar{c}}_{i}\right),\;\;L_{\left(2i+1)(2i\right)}=\cos\left(\Lambda {\bar{c}}_{i}\right),\;\\ L_{\left(2i+1)(2i+1\right)}=\sin\left(\Lambda {\bar{c}}_{i}\right),\;\;L_{\left(2i+1)(2i+2\right)}=-\cos\left(\Lambda {\bar{c}}_{i}\right),\;\\ L_{\left(2i+2)(2i-1\right)}={\bar{k}}_{i}\cos\left(\Lambda {\bar{c}}_{i}\right)-\varpi \sqrt{1+\chi_{1}^{2}}\;\\ \;\;\;\;\;\;\;\;\;\;\;\;\;\;\;\;\;\times \sqrt{\left(1+\chi_{2}^{2}\right)+\left(1+\chi_{3}^{2}\right)\left({\bar{H}}_{y}^{2}+{\bar{H}}_{z}^{2}\right)-\varpi^{2}\mu^{2}\left(1+\chi_{1}^{2}\right)}\;\sin\left(\Lambda {\bar{c}}_{i}\right),\;\\ L_{\left(2i+2)(2i\right)}={\bar{k}}_{i}\sin\left(\Lambda {\bar{c}}_{i}\right)+\varpi \sqrt{1+\chi_{1}^{2}}\;\\ \;\;\;\;\;\;\;\;\;\;\;\;\;\;\;\;\;\times \sqrt{\left(1+\chi_{2}^{2}\right)+\left(1+\chi_{3}^{2}\right)\left({\bar{H}}_{y}^{2}+{\bar{H}}_{z}^{2}\right)-\varpi^{2}\mu^{2}\left(1+\chi_{1}^{2}\right)}\;\cos\left(\Lambda {\bar{c}}_{i}\right),\;\\ L_{\left(2i+2)(2i+1\right)}=-{\bar{k}}_{i}\cos\left(\Lambda {\bar{c}}_{i}\right),\;\;L_{\left(2i+2)(2i+2\right)}=-{\bar{k}}_{i}\sin\left(\Lambda {\bar{c}}_{i}\right), \end{array}$$

and in the case of the FIFR condition, the nonvanishing elements of L are given by:

(A2) $$\begin{array}{c} L_{11}=1,\;\;L_{2(2N+1)}=-\sin\left(\Lambda \right),\;\;L_{2(2N+2)}=\cos\left(\Lambda \right),\\ L_{\left(2i+1)(2i-1\right)}=-\sin\left(\Lambda {\bar{c}}_{i}\right),\;\;L_{\left(2i+1)(2i\right)}=\cos\left(\Lambda {\bar{c}}_{i}\right),\;\\ L_{\left(2i+1)(2i+1\right)}=\sin\left(\Lambda {\bar{c}}_{i}\right),\;\;L_{\left(2i+1)(2i+2\right)}=-\cos\left(\Lambda {\bar{c}}_{i}\right),\;\\ L_{\left(2i+2)(2i-1\right)}={\bar{k}}_{i}\cos\left(\Lambda {\bar{c}}_{i}\right)-\varpi \sqrt{1+\chi_{1}^{2}}\;\\ \;\;\;\;\;\;\;\;\;\;\;\;\;\;\;\;\;\times \sqrt{\left(1+\chi_{2}^{2}\right)+\left(1+\chi_{3}^{2}\right)\left({\bar{H}}_{y}^{2}+{\bar{H}}_{z}^{2}\right)-\varpi^{2}\mu^{2}\left(1+\chi_{1}^{2}\right)}\;\sin\left(\Lambda {\bar{c}}_{i}\right),\;\\ L_{\left(2i+2)(2i\right)}={\bar{k}}_{i}\sin\left(\Lambda {\bar{c}}_{i}\right)+\varpi \sqrt{1+\chi_{1}^{2}}\;\\ \;\;\;\;\;\;\;\;\;\;\;\;\;\;\;\;\;\times \sqrt{\left(1+\chi_{2}^{2}\right)+\left(1+\chi_{3}^{2}\right)\left({\bar{H}}_{y}^{2}+{\bar{H}}_{z}^{2}\right)-\varpi^{2}\mu^{2}\left(1+\chi_{1}^{2}\right)}\;\cos\left(\Lambda {\bar{c}}_{i}\right),\;\\ L_{\left(2i+2)(2i+1\right)}=-{\bar{k}}_{i}\cos\left(\Lambda {\bar{c}}_{i}\right),\;\;L_{\left(2i+2)(2i+2\right)}=-{\bar{k}}_{i}\sin\left(\Lambda {\bar{c}}_{i}\right). \end{array}$$

## Appendix B. Frequencies and Vibration Modes of a Magnetically Affected Nanorod with a Single Defect

Using Equation (23), the amplitude functions of the doubly constitutive portions of the locally defected nanorod under action of the transverse magnetic field are given by: ${\bar{U}}_{1}\left(\xi \right)=A_{1}\cos\left(\Lambda \xi \right)+B_{1}\sin\left(\Lambda \xi \right)$ and ${\bar{U}}_{2}\left(\xi \right)=A_{2}\cos\left(\Lambda \xi \right)+B_{2}\sin\left(\Lambda \xi \right)$. In the following parts, we proceed in analytical solution of the free vibration of the problem for both FIFI and FIFR ends.

### Appendix B.1. FIFI Boundary Conditions

Through satisfying the given conditions in Equations (21a), (21b), and (22a), the following set of algebraic equations is gained:

(A3) $$\begin{array}{l} \begin{array}{c} \left(\begin{array}{cccc} 1 & 0 & 0 & 0\\ 0 & 0 & \cos\left(\Lambda \right) & \sin\left(\Lambda \right)\\ \left(\begin{array}{c} {\bar{k}}_{1}\cos\left(\Lambda {\bar{c}}_{1}\right)-\varpi \sqrt{1+\chi_{1}^{2}}\\ \sqrt{\left(\begin{array}{c} \left(1+\chi_{2}^{2}\right)+\\ \left(1+\chi_{3}^{2}\right)\left({\bar{H}}_{y}^{2}+{\bar{H}}_{z}^{2}\right)\\ -\mu^{2}\varpi^{2}\left(1+\chi_{1}^{2}\right) \end{array}\right)}\sin\left(\Lambda {\bar{c}}_{1}\right) \end{array}\right) & \left(\begin{array}{c} {\bar{k}}_{1}\sin\left(\Lambda {\bar{c}}_{1}\right)+\varpi \sqrt{1+\chi_{1}^{2}}\\ \sqrt{\left(\begin{array}{c} \left(1+\chi_{2}^{2}\right)+\\ \left(1+\chi_{3}^{2}\right)\left({\bar{H}}_{y}^{2}+{\bar{H}}_{z}^{2}\right)\\ -\mu^{2}\varpi^{2}\left(1+\chi_{1}^{2}\right) \end{array}\right)}\cos\left(\Lambda {\bar{c}}_{1}\right) \end{array}\right) & -{\bar{k}}_{1}\cos\left(\Lambda {\bar{c}}_{1}\right) & -k_{1}\sin\left(\Lambda {\bar{c}}_{1}\right)\\ -\Lambda \sin\left(\Lambda {\bar{c}}_{1}\right) & \Lambda \cos\left(\Lambda {\bar{c}}_{1}\right) & \Lambda \sin\left(\Lambda {\bar{c}}_{1}\right) & -\Lambda \cos\left(\Lambda {\bar{c}}_{1}\right) \end{array}\right) \end{array}\\ \times \left(\begin{array}{c} A_{1}\\ B_{1}\\ A_{2}\\ B_{2} \end{array}\right)=\left(\begin{array}{c} 0\\ 0\\ 0\\ 0 \end{array}\right). \end{array}$$

A solution to Equation (A3) can be sought by setting the coefficient matrix determinant equal to zero. By doing so, the dispersion relation (i.e., characteristic equation) takes the following form:

(A4) $$\begin{array}{c} 2{\bar{k}}_{1}\sin\left(\frac{\varpi \;\sqrt{1+\chi_{1}^{2}}}{\sqrt{\left(1+\chi_{2}^{2}\right)+\left(1+\chi_{3}^{2}\right)\left({\bar{H}}_{y}^{2}+{\bar{H}}_{z}^{2}\right)-\mu^{2}\varpi^{2}\left(1+\chi_{1}^{2}\right)}}\right)\;\\ +\left(\varpi \sqrt{1+\chi_{1}^{2}}\;\sqrt{\left(1+\chi_{2}^{2}\right)+\left(1+\chi_{3}^{2}\right)\left({\bar{H}}_{y}^{2}+{\bar{H}}_{z}^{2}\right)-\mu^{2}\varpi^{2}\left(1+\chi_{1}^{2}\right)}\right)\;\\ \times \left[\cos\left(\frac{\varpi \;\sqrt{1+\chi_{1}^{2}}}{\sqrt{\left(1+\chi_{2}^{2}\right)+\left(1+\chi_{3}^{2}\right)\left({\bar{H}}_{y}^{2}+{\bar{H}}_{z}^{2}\right)-\mu^{2}\varpi^{2}\left(1+\chi_{1}^{2}\right)}}\right)+\right.\;\\ \left.\cos\left(\frac{\varpi \left(2{\bar{c}}_{1}-1\right)\;\sqrt{1+\chi_{1}^{2}}}{\sqrt{\left(1+\chi_{2}^{2}\right)+\left(1+\chi_{3}^{2}\right)\left({\bar{H}}_{y}^{2}+{\bar{H}}_{z}^{2}\right)-\mu^{2}\varpi^{2}\left(1+\chi_{1}^{2}\right)}}\right)\right]=0, \end{array}$$

and the vibration mode shapes (i.e., amplitude functions) are stated by:

(A5a) $$\begin{array}{c} \begin{array}{c} {\bar{U}}_{1}\left(\xi \right)=B_{1}\sin\left(\frac{\varpi \;\sqrt{1+\chi_{1}^{2}}\;\xi }{\sqrt{\left(1+\chi_{2}^{2}\right)+\left(1+\chi_{3}^{2}\right)\left({\bar{H}}_{y}^{2}+{\bar{H}}_{z}^{2}\right)-\mu^{2}\varpi^{2}\left(1+\chi_{1}^{2}\right)}}\right), \end{array} \end{array}$$

(A5b) $$\begin{array}{c} \begin{array}{c} {\bar{U}}_{2}\left(\xi \right)=A_{2}\sin\left(\frac{\varpi \;\sqrt{1+\chi_{1}^{2}}\;\left(1-\xi \right)}{\sqrt{\left(1+\chi_{2}^{2}\right)+\left(1+\chi_{3}^{2}\right)\left({\bar{H}}_{y}^{2}+{\bar{H}}_{z}^{2}\right)-\mu^{2}\varpi^{2}\left(1+\chi_{1}^{2}\right)}}\right). \end{array} \end{array}$$

In the particular case of the removal of the local defect from the nanorod (i.e., k→∞), the nonlocal-surface energetic-natural frequencies of the FIFI nanorod in the dimensionless form are evaluated from Equation (A4) as follows:

(A6) $$\begin{array}{c} \varpi_{m}^{snl}=\frac{m\pi \sqrt{\left(1+\chi_{2}^{2}\right)+\left(1+\chi_{3}^{2}\right)\left({\bar{H}}_{y}^{2}+{\bar{H}}_{z}^{2}\right)}}{\sqrt{\left(1+\chi_{1}^{2}\right)\left(1+{\left(m\pi \mu \right)}^{2}\right)}};\;\;m=1,2,\ldots \end{array}$$

and by ignoring both nonlocality and surface effects, the dimensionless-classical natural frequencies of the perfect nanorod are readily given by: $\varpi_{m}^{cl}=m\pi$. To show the roles of these effects on the classical natural frequencies, the frequency ratio (FR) factor is defined by:

(A7) $$\begin{array}{c} \mathrm{FR}=\frac{\varpi_{m}^{snl}}{\varpi_{m}^{cl}}=\frac{\sqrt{\left(1+\chi_{2}^{2}\right)+\left(1+\chi_{3}^{2}\right)\left({\bar{H}}_{y}^{2}+{\bar{H}}_{z}^{2}\right)}}{\sqrt{\left(1+{\left(m\pi \mu \right)}^{2}\right)\left(1+\chi_{1}^{2}\right)}}. \end{array}$$

### Appendix B.2. FIFR Boundary Conditions

By enforcing the conditions in Equations (21a), (21b), and (22b),

(A8) $$\begin{array}{c} \left(\begin{array}{cccc} 1 & 0 & 0 & 0\\ 0 & 0 & -\Lambda sin\left(\Lambda \right) & \Lambda \cos\left(\Lambda \right)\\ \left(\begin{array}{c} {\bar{k}}_{1}\sin\left(\Lambda {\bar{c}}_{1}\right)-\varpi \sqrt{1+\chi_{1}^{2}}\\ \sqrt{\left(\begin{array}{c} \left(1+\chi_{2}^{2}\right)+\\ \left(1+\chi_{3}^{2}\right)\left({\bar{H}}_{y}^{2}+{\bar{H}}_{z}^{2}\right)\\ -\mu^{2}\varpi^{2}\left(1+\chi_{1}^{2}\right) \end{array}\right)}\cos\left(\Lambda {\bar{c}}_{1}\right) \end{array}\right) & \left(\begin{array}{c} {\bar{k}}_{1}\sin\left(\Lambda {\bar{c}}_{1}\right)+\varpi \sqrt{1+\chi_{1}^{2}}\\ \sqrt{\left(\begin{array}{c} \left(1+\chi_{2}^{2}\right)+\\ \left(1+\chi_{3}^{2}\right)\left({\bar{H}}_{y}^{2}+{\bar{H}}_{z}^{2}\right)\\ -\mu^{2}\varpi^{2}\left(1+\chi_{1}^{2}\right) \end{array}\right)}\cos\left(\Lambda {\bar{c}}_{1}\right) \end{array}\right) & -{\bar{k}}_{1}\cos\left(\Lambda {\bar{c}}_{1}\right) & -{\bar{k}}_{1}\sin\left(\Lambda {\bar{c}}_{1}\right)\\ -\Lambda \sin\left(\Lambda {\bar{c}}_{1}\right) & \Lambda \cos\left(\Lambda {\bar{c}}_{1}\right) & \Lambda \sin\left(\Lambda {\bar{c}}_{1}\right) & -\Lambda \cos\left(\Lambda {\bar{c}}_{1}\right) \end{array}\right)\\ \times \left(\begin{array}{c} A_{1}\\ B_{1}\\ A_{2}\\ B_{2} \end{array}\right)=\left(\begin{array}{c} 0\\ 0\\ 0\\ 0 \end{array}\right). \end{array}$$

The requirement of non-zero free dynamic response leads to the dispersion relation of the magnetically affected FIFR nanorod with a locally defected zone as follows:

(A9) $$\begin{array}{c} {\bar{k}}_{1}\cos\left(\frac{\varpi \;\sqrt{1+\chi_{1}^{2}}}{\sqrt{\left(1+\chi_{2}^{2}\right)+\left(1+\chi_{3}^{2}\right)\left({\bar{H}}_{y}^{2}+{\bar{H}}_{z}^{2}\right)-\mu^{2}\varpi^{2}\left(1+\chi_{1}^{2}\right)}}\right)-\;\\ \left(\varpi \sqrt{1+\chi_{1}^{2}}\sqrt{\left(1+\chi_{2}^{2}\right)+\left(1+\chi_{3}^{2}\right)\left({\bar{H}}_{y}^{2}+{\bar{H}}_{z}^{2}\right)-\mu^{2}\varpi^{2}\left(1+\chi_{1}^{2}\right)}\right)\;\\ \times \cos\left(\frac{\varpi \;{\bar{c}}_{1}\;\sqrt{1+\chi_{1}^{2}}}{\sqrt{\left(1+\chi_{2}^{2}\right)+\left(1+\chi_{3}^{2}\right)\left({\bar{H}}_{y}^{2}+{\bar{H}}_{z}^{2}\right)-\mu^{2}\varpi^{2}\left(1+\chi_{1}^{2}\right)}}\right)\;\\ \sin\left(\frac{\varpi \;\left(1-{\bar{c}}_{1}\right)\;\sqrt{1+\chi_{1}^{2}}}{\sqrt{\left(1+\chi_{2}^{2}\right)+\left(1+\chi_{3}^{2}\right)\left({\bar{H}}_{y}^{2}+{\bar{H}}_{z}^{2}\right)-\mu^{2}\varpi^{2}\left(1+\chi_{1}^{2}\right)}}\right)=0, \end{array}$$

and the corresponding mode shapes are written as:

(A10a) $$\begin{array}{c} \begin{array}{c} {\bar{U}}_{1}\left(\xi \right)=B_{1}\sin\left(\frac{\varpi \;\sqrt{1+\chi_{1}^{2}}\;\xi }{\sqrt{\left(1+\chi_{2}^{2}\right)+\left(1+\chi_{3}^{2}\right)\left({\bar{H}}_{y}^{2}+{\bar{H}}_{z}^{2}\right)-\mu^{2}\varpi^{2}\left(1+\chi_{1}^{2}\right)}}\right), \end{array} \end{array}$$

(A10b) $$\begin{array}{c} \begin{array}{c} {\bar{U}}_{2}\left(\xi \right)=A_{2}\;\cos\left(\frac{\varpi \;\sqrt{1+\chi_{1}^{2}}\;\left(1-\xi \right)}{\sqrt{\left(1+\chi_{2}^{2}\right)+\left(1+\chi_{3}^{2}\right)\left({\bar{H}}_{y}^{2}+{\bar{H}}_{z}^{2}\right)-\mu^{2}\varpi^{2}\left(1+\chi_{1}^{2}\right)}}\right) \end{array} \end{array}$$

As a particular case, for a perfect nanorod (i.e., $k_{1}\to \infty$), the dimensionless natural frequencies for the FIFR condition are calculated from Equation (A9) as:

(A11) $$\begin{array}{c} \varpi_{m}^{snl}=\frac{\left(2m-1\right)\frac{\pi }{2}\sqrt{\left(1+\chi_{2}^{2}\right)+\left(1+\chi_{3}^{2}\right)\left({\bar{H}}_{y}^{2}+{\bar{H}}_{z}^{2}\right)}}{\sqrt{\left(1+\chi_{1}^{2}\right)\left(1+{\left(\frac{\pi \mu \left(2m-1\right)}{2}\right)}^{2}\right)}}, \end{array}$$

and the frequency ratio is given by:

(A12) $$\begin{array}{c} \mathrm{FR}=\frac{\varpi_{m}^{snl}}{\varpi_{m}^{cl}}=\frac{\sqrt{\left(1+\chi_{2}^{2}\right)+\left(1+\chi_{3}^{2}\right)\left({\bar{H}}_{y}^{2}+{\bar{H}}_{z}^{2}\right)}}{\sqrt{\left(1+{\left(\frac{\pi \mu \left(2m-1\right)}{2}\right)}^{2}\right)\left(1+\chi_{1}^{2}\right)}}. \end{array}$$
